# Genome Analysis Reveals Interplay between 5′UTR Introns and Nuclear mRNA Export for Secretory and Mitochondrial Genes

**DOI:** 10.1371/journal.pgen.1001366

**Published:** 2011-04-14

**Authors:** Can Cenik, Hon Nian Chua, Hui Zhang, Stefan P. Tarnawsky, Abdalla Akef, Adnan Derti, Murat Tasan, Melissa J. Moore, Alexander F. Palazzo, Frederick P. Roth

**Affiliations:** 1 Department of Biological Chemistry and Molecular Pharmacology, Harvard Medical School, Boston, Massachusetts, United States of America; 2 Department of Biochemistry, University of Toronto, Toronto, Canada; 3 Department of Biochemistry and Molecular Pharmacology, Howard Hughes Medical Institute, University of Massachusetts Medical School, Worcester, Massachusetts, United States of America; 4 Center for Cancer Systems Biology, Dana-Farber Cancer Institute, Boston, Massachusetts, United States of America; 5 Donnelly Centre for Cellular and Biomolecular Research, University of Toronto, Toronto, Canada; 6 Samuel Lunenfeld Research Institute, Mt. Sinai Hospital, Toronto, Canada; Stanford University School of Medicine, United States of America

## Abstract

In higher eukaryotes, messenger RNAs (mRNAs) are exported from the nucleus to the cytoplasm via factors deposited near the 5′ end of the transcript during splicing. The signal sequence coding region (SSCR) can support an alternative mRNA export (ALREX) pathway that does not require splicing. However, most SSCR–containing genes also have introns, so the interplay between these export mechanisms remains unclear. Here we support a model in which the furthest upstream element in a given transcript, be it an intron or an ALREX–promoting SSCR, dictates the mRNA export pathway used. We also experimentally demonstrate that nuclear-encoded mitochondrial genes can use the ALREX pathway. Thus, ALREX can also be supported by nucleotide signals within mitochondrial-targeting sequence coding regions (MSCRs). Finally, we identified and experimentally verified novel motifs associated with the ALREX pathway that are shared by both SSCRs and MSCRs. Our results show strong correlation between 5′ untranslated region (5′UTR) intron presence/absence and sequence features at the beginning of the coding region. They also suggest that genes encoding secretory and mitochondrial proteins share a common regulatory mechanism at the level of mRNA export.

## Author Summary

The function and evolution of introns have been topics of great interest since introns were discovered in the 1970s. Introns that interrupt protein-coding regions have the most obvious potential to affect coding sequences and their evolution, and they have therefore been studied most intensively. However, about one third of human genes contain introns within 5′ untranslated regions (UTR). Here we observe that certain classes of genes, including those targeted to the endoplasmic reticulum and nuclear-encoded mitochondrial genes, are surprisingly depleted of 5′UTR introns. We offer and support a model that explains this observation and points to a surprising connection between 5′UTR introns and how mRNAs are exported from the nucleus.

## Introduction

In humans, ∼35% of all genes have introns in their 5′ untranslated regions (UTRs) [1]–[3]. These introns differ from those in coding regions, for example, in typical length and nucleotide composition [1]–[3]. Previously, 5′UTR introns (5UIs) were suggested to be evolving under a neutral model of random insertion and deletion events with the sole constraint of avoiding upstream open reading frames [3]. Recently, we showed that presence and length of 5UIs correlates with the level of expression across cells and tissue types [1]. More importantly, we observed an uneven distribution of 5UIs amongst genes across specific functional categories [1]. Genes with regulatory roles, including non-receptor tyrosine kinases, regulators of cytoskeleton, transcription and metabolism, were enriched in having 5UIs [1]. Our results suggested that many 5UIs are evolving under complex selective forces as opposed to a simple model of neutral evolution [1]. However, it is unclear whether there is any widely used mode of regulation that is unique to 5UIs.

In eukaryotes, splicing is coupled to key mRNA metabolic processes. During the act of splicing, several different protein complexes are deposited onto mRNA. For example, the Transcription Export (TREX) complex promotes the nuclear export of fully processed transcripts [4]. In higher eukaryotes, the TREX complex is deposited primarily onto the 5′ end of nascent transcripts by the cooperative action of the cap-binding complex and the spliceosome [5]. Given that 5UIs are necessarily proximal to 5′ ends of transcripts, an intriguing possibility is that splicing of 5UIs could have a disproportionate impact on mRNA export by promoting TREX recruitment. Although the majority of transcripts follow the splicing-dependent export pathway, alternative pathways exist. Recently, Palazzo *et al.* demonstrated that mRNAs that encode secreted proteins can use an alternative route for mRNA export that is mediated by a nucleotide element within the signal sequence coding region (SSCR) [6]. In contrast to the splicing-dependent pathway, this alternative RNA export (ALREX) pathway does not require splicing or a 5′ cap [6]. Vertebrate SSCRs were found to be adenine-poor and silent mutations introducing adenines into the SSCR impair its ability to promote mRNA export [6]. However, beyond adenine-depletion this element has been poorly characterized. Furthermore, it has remained unclear which SSCR-containing transcripts use ALREX and to what extent, since the vast majority of SSCR-containing transcripts are also spliced and thus could potentially use the canonical export pathway. The fact that both ALREX signals and splicing signals are found near the 5′ end of genes, suggests the interesting possibility that competition between signals at the 5′end of transcripts determines how a given mRNA is exported.

Here, we extend our computational analysis of 5UIs to identify functional groups of genes that preferentially lack these introns. We find that 5UIs are depleted in genes containing SSCRs or mitochondrial-targeting sequence coding regions (MSCRs). We demonstrate that SSCRs and MSCRs derived from 5UI-lacking (5UI^−^) genes contain sequence features associated with ALREX and promote export *in vivo*. In stark contrast, SSCRs and MSCRs derived from 5UI^+^ genes do not exhibit ALREX-associated features. Furthermore, we show that 5UI^+^ genes do not support splicing-independent mRNA export. We then characterize ALREX elements more fully by identifying and validating new ALREX-associated motifs. Taken together, our results support a model wherein the 5′-most element in a newly synthesized transcript, be it an intron or an ALREX element, dictates which pathway is employed for export. Furthermore, our results provide the first known regulatory role that is unique to 5′ UTR introns and suggest that it is widely used.

## Results

### SSCR– and MSCR–containing genes are depleted of introns in their 5′UTRs

Using a high quality set of 5UI definitions for human, we observed a depletion of 5UIs amongst genes with certain Gene Ontology [7] (GO) annotations (Table S1). Examples of 5UI-depleted GO terms include “MHC class II protein complex” (ratio of 5UI-containing genes to total genes annotated with particular GO term is 0/25), “aspartic endopeptidase activity” (0/23), “voltage-gated calcium channel activity” (2/35), “growth factor activity” (33/180), “electron carrier activity” (27/145), and “extracellular space” (108/497). In each case, these ratios are significantly lower than the ratio of ∼35% expected by chance (p<0.05 after adjusting for multiple hypothesis testing). More generally, we observed a depletion of 5UIs among nuclear genes encoding three protein classes.

The first class was composed of protein families encoded by mostly intronless genes. This group includes histone genes [8], olfactory receptors, G-protein coupled receptors [9], and keratins [10], [11]. Depletion of 5UIs in these gene classes does not suggest any 5UI-specific phenomena, as these genes are more generally intron-depleted.

**Figure 1:**
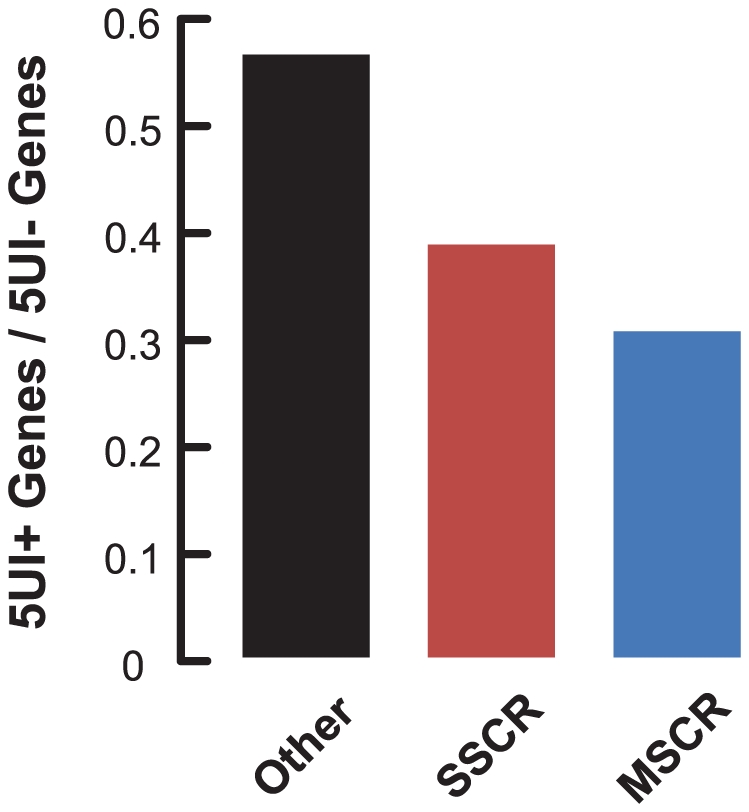
5′UTR introns (5UIs) are depleted in genes that contain SSCRs and MSCRs. Fraction of genes with 5UIs was plotted for SSCR, MSCR or other human protein coding genes.

The second class was composed of secreted or membrane-bound proteins that are trafficked through the endoplasmic reticulum (ER). We compiled a list of all genes with signal sequence coding regions (SSCRs), encoding N-terminal cleavable signal sequence peptides that target newly synthesized proteins to the ER [12] (see Materials and Methods). We observed that 5UIs were generally depleted among SSCR-containing genes (Figure 1; Fisher's Exact Test *p* = 8×10^-8^, odds ratio 0.84).

The last class included proteins localized to mitochondria. Nuclear-encoded mitochondrial genes are translated in the cytoplasm and are targeted via an N-terminal leader peptide sequence to mitochondria [13], [14]. We compiled a list of genes with mitochondrial-targeting sequence coding regions (MSCRs), and observed that 5UIs were depleted in MSCR-containing genes (Figure 1; Fisher's Exact Test *p* = 8×10^-6^; odds ratio 0.59). This depletion is even stronger than that observed for SSCR-containing genes. Thus, our results showed a general depletion of 5UIs among genes encoding either ER-targeted or mitochondrial proteins.

Next, we tested whether 5UI depletion in SSCR or MSCR-containing genes is a secondary effect of these genes having short 5′UTRs. Although 5UIs are more likely amongst genes with long 5′UTRs (Figure S1A, Wilcoxon Rank Sum Test p <2×10^-16^; a 99 nt greater median 5′UTR length in 5UI^+^ than 5UI^−^ genes), we observed that genes encoding secreted and mitochondrial proteins have 5′UTRs that are only slightly shorter than other genes (Figure S1B, Wilcoxon Rank Sum Test p =  2×10^-15^, p = 9×10^-9^; a 25 nt and 51 nt difference in median 5′UTR length for SSCR- and MSCR-containing genes, respectively). Even after correcting for the differences in 5′UTR length, SSCR- and MSCR-containing genes were significantly depleted of 5UIs (see Text S1). Similarly, the depletion of 5UIs did not reflect an overall decrease in intronic content, as the total number of bases in non-5′UTR introns did not differ between genes containing or lacking SSCRs (Welch Two Sample t-test, *p* = 0.34; Figure S2).

### SSCRs from 5UI^−^ genes, but not 5UI^+^ genes, promote ALREX

A possible link between splicing and genes encoding secretory proteins is the nuclear export of mRNA. Several studies have indicated that export factors are loaded near the 5′ cap co-transcriptionally during the splicing of the more 5′-proximal intron [5], [15]. SSCRs, which similarly promote mRNA export via ALREX [6], are located at the 5′end of the open reading frame (ORF) and could also potentially be recognized by factors co-transcriptionally. Hence, we hypothesized that the 5′-most element in a given transcript, be it an intron or an SSCR, dictates the pathway by which that transcript is exported.

**Figure 2:**
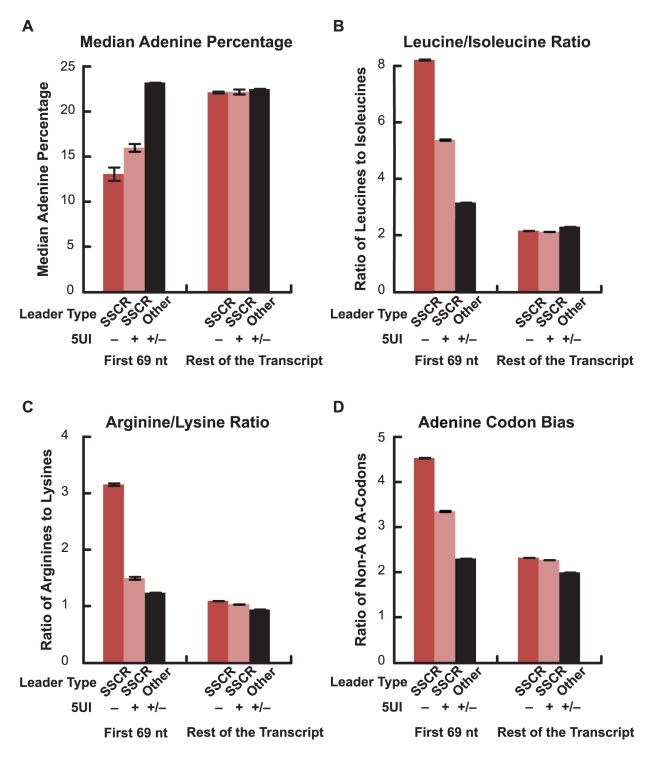
Adenine depletion in SSCRs is attenuated by 5UIs. All bars represent the average from the first 69 nucleotides of all SSCRs-containing open reading frames from the human genome which either lacked (“−“) or had (“+”) 5UIs. As controls, these sequences were compared to the rest of the open reading frame, or analogous regions from open reading frames that lacked SSCRs (“other”). Error bars correspond to standard errors of the median or mean, as appropriate. (A) Median adenine percentage. Standard error of the median was determined by bootstrap-resampling. (B) The mean ratio between the number of encoded leucines to isoleucines. (C) The mean ratio between the number of encoded arginines to lysines. (D) The ratio of adenine- lacking, to adenine-containing codons for all amino acids that have both types of codons (L, V, A, P, S, G, and R). See Figure S3 for examples of adenine codon bias for specific amino acids (leucine and serine).

Signal peptide sequences contain a hydrophobic core with amino acids that are naturally encoded by codons with low adenine content. In addition, for pairs of biochemically similar amino acids that differ in the adenine content of their corresponding codons, SSCRs tend to prefer the amino acid with low adenine content codons [6]. We previously showed that adenine depletion in SSCRs is functionally linked to ALREX as silent adenine mutations partially inhibit ALREX [6]. Our hypothesis of a competition between export pathways, driven by whether the 5′-most element is a 5UI or an ALREX signal, predicts that the selection pressure to maintain sequence features important for ALREX-dependent mRNA export would be relaxed in transcripts with 5UIs. We therefore tested whether adenine depletion in SSCRs is attenuated in genes containing 5UIs. Remarkably, we found that SSCRs from genes lacking 5UIs contain 18.2% fewer adenines when compared to SSCRs from genes carrying 5UIs (Figure 2A; Wilcoxon Rank Sum Test *p* = 4×10^-49^). Next, we analyzed the amino acid preference of SSCR-containing genes for pairs of biochemically similar amino acids. Specifically, we observed that SSCRs of 5UI^−^ genes have a significantly increased ratio of leucine (which has adenine-poor codons) to isoleucine (which has at least one adenine in all of its three codons) and of arginine (with relatively adenine-poor codons) relative to lysine as compared to SSCRs of 5UI^+^ genes (Figure 2B–2C; Fisher's Exact Test, *p* = 3×10^-27^ and 3×10^-40^, 95% confidence interval of odds ratio 1.4–1.7 and 1.9–2.4 respectively). SSCRs also exhibit a bias towards synonymous codons that lack adenine [6]. Importantly, this bias diminishes for 5UI^+^ genes (Figure 2D). This was true for codons for any given single amino acid, such as leucine or serine (Figure S3), or when all synonymous codons were aggregated (Figure 2D; Fisher's Exact Test *p* = 2×10^-42^; 95% CI of odds ratio 1.3-1.4). Taken together, our computational analysis indicates that the bias of SSCRs against adenines is relaxed in 5UI^+^ genes. Furthermore, this reduced bias appears to be due to a relaxation of nucleotide-level constraints, supporting the idea that the presence of 5UIs relieves selection maintaining ALREX signals.

**Figure 3:**
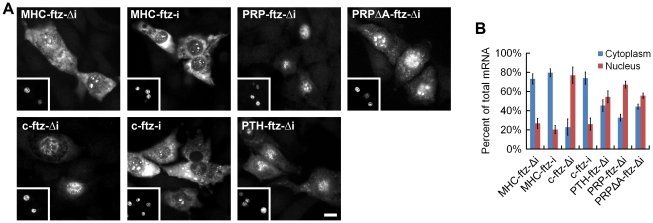
SSCRs derived from 5UI^−^ genes promote mRNA nuclear export. Capped and polyadenylated transcripts encoding various versions of *ftz* (see for all modified *ftz* sequences) were microinjected along with fluorescent 70 kD dextran into the nuclei of NIH 3T3 fibroblasts. After 1 hour, samples were fixed and mRNA was detected by FISH against *ftz*. (A) Representative micrographs of the mRNA distribution and fluorescent 70 kD dextran (inserts). Scale Bar = 15 µm. (B) Quantification of the cytoplasmic over the total fluorescence signal. Each bar represents an average of three experiments, each of which consisted of 15–30 cells. Error bars represent the standard deviation between the three experiments.

To experimentally investigate this intriguing connection between sequence features in the coding region and the presence or absence of 5UIs, we tested whether SSCRs derived from genes with 5UIs are defective in promoting mRNA export. We inserted SSCR elements into a fragment of the fushi tarazu (*ftz*), just downstream of the start codon. Furthermore we generated versions of *ftz* that either contained (*ftz-i*) or lacked (*ftz-Δ*i) its endogenous intron. Modified forms of these transcripts were previously used to study splicing- and SSCR-dependent mRNA nuclear export [6], [16]. Polyadenylated forms of the *ftz* mRNA were microinjected into the nuclei of NIH 3T3 mouse fibroblasts. After incubating the cells for one hour, mRNA export was visually monitored by fluorescence *in situ* hybridization (FISH, Figure 3A) and the amount of mRNA nuclear export was quantified (Figure 3B). Nuclear injection was confirmed by co-injecting fluorescently labeled 70 kD dextran, which is too large to passively diffuse through nuclear pores (see insets, Figure 3A). As demonstrated by several groups, we found that a version of the *ftz* mRNA that encodes a cytoplasmic protein, but contains neither an intron nor an SSCR (*c-ftz-Δi*), was not efficiently exported [6], [16] (Figure 3). Nuclear export could be rescued if an intron was incorporated (*c-ftz-i*). As reported previously, SSCRs from the *MHC* class 2 gene *H2-k1*, which lacks a 5UI, promoted efficient export of an intronless version of *ftz* (Figure 3, *MHC-ftz-Δi*; see Palazzo *et al.* [6] and Figure S4 for all *ftz* variant sequences). We next examined the *parathyroid hormone* (*PTH*) and the *prion protein* (*PRP*) SSCRs, both derived from genes with 5UIs. Consistent with trends we observed for 5UI^+^ genes in general, neither *PTH* nor *PRP* SSCRs are depleted in adenine content. Furthermore, neither promoted efficient export (Figure 3, *PTH-ftz-Δi* and *PRP-ftz-Δi*). Interestingly, elimination of adenines from the *PRP* SSCR (*PRPΔA*) only marginally stimulated export (Figure 3, *PRPΔA-ftz-Δi*) suggesting that this SSCR lacks other features crucial for stimulating export. In summary, only SSCRs from genes lacking 5UIs promoted efficient mRNA export, experimentally demonstrating a functional relevance for the computationally-discovered connection between coding sequence features and 5UI status.

### MSCRs from 5UI^−^ genes, but not 5UI^+^ genes, promote ALREX

**Figure 4:**
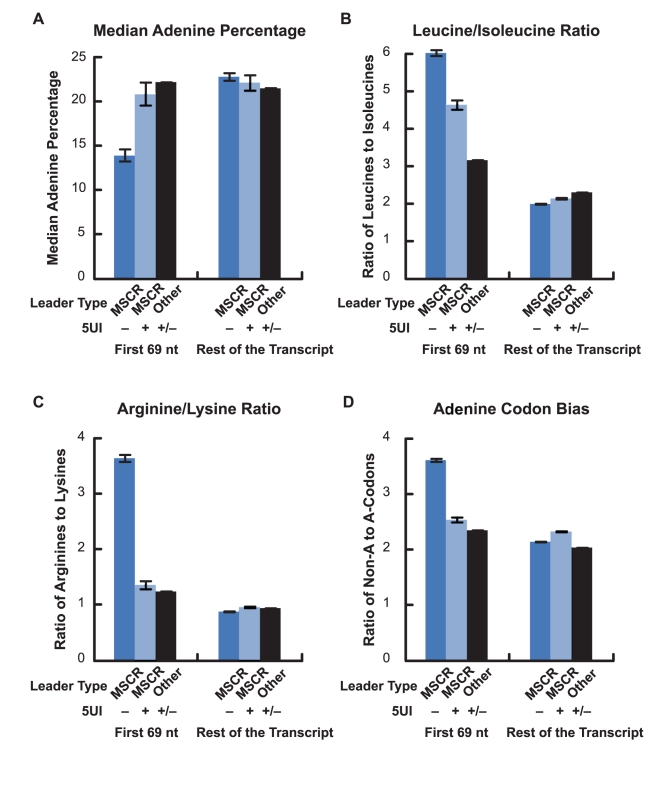
Adenine depletion in MSCRs derived from 5UI^−^ genes. As in Figure 2A, all bars represent the average from the first 69 nucleotides of all MSCRs-containing open reading frames from the human genome which either lacked (“–“) or had (“+”) 5UIs. As controls these sequences were compared to the rest of the open reading frame, or analogous regions from open reading frames that lacked SSCRs (“other”). Error bars correspond to the standard error of the median or mean, as appropriate. Panels A-D are as described for Figure 2. See Figure S3 for examples of adenine codon bias for specific amino acids (leucine and serine).

Our investigation into the relationship between 5UIs and alternative export began with the observation that 5UIs were depleted amongst secretory genes. Because 5UIs are also depleted amongst nuclear-encoded mitochondrial genes (Figure 1), we wondered whether related phenomena might be at play. Like secreted proteins, mitochondrial proteins contain a cleavable leader peptide that dictates the ultimate localization of the polypeptide chain [13], [14]. We therefore wondered whether MSCRs exhibit the same nucleotide features that had been associated with ALREX in SSCRs. Indeed MSCRs, like SSCRs, were depleted in adenines overall. Also like SSCRs, this adenine depletion was restricted to MSCRs derived from 5UI^−^ genes (Figure 4A; Wilcoxon Rank Sum Test *p* =  2×10^-9^). We found that MSCRs, like SSCRS, tend to encode leucine relative to isoleucine (Figure 4B), and arginine relative to lysine (Figure 4C). Just as with SSCRs, this phenomenon was more pronounced when the elements were derived from 5UI^−^ genes (Fisher's Exact Test *p* = 0.16 and 10^−9^, 95% CI of odds ratio 0.9–1.9 and 1.9–3.7 respectively). Finally, only MSCRs from 5UI^−^ genes displayed a bias for synonymous codons that lacked adenine (Figure 4D). This was true for codons coding for any given single amino acid examined, such as leucine or serine (Figure S3), or when results for all synonymous codons were aggregated (Figure 4D; Fisher's Exact Test *p* = 7×10^-06^; 95% CI for odds ratio 1.2-1.7).

**Figure 5:**
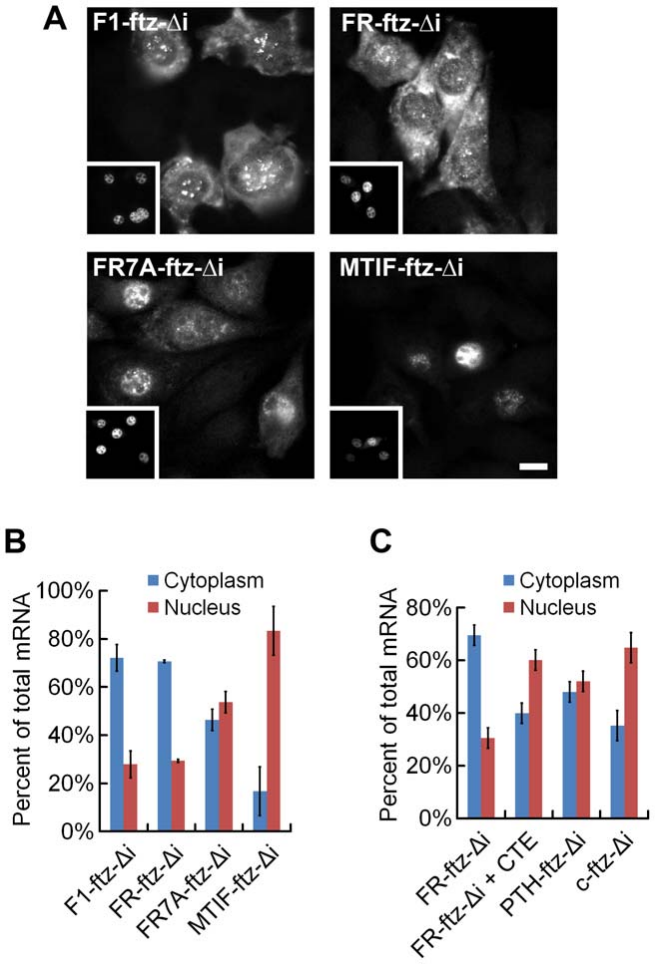
MSCRs derived from 5UI^−^ genes promote mRNA nuclear export. Transcripts encoding various versions of *ftz* (see Figure S4 for all modified *ftz* sequences) were microinjected along with fluorescent 70 kD dextran as in Figure 3. Panels A and B are as described for Figure 3. (C) DNA plasmids containing the indicated *ftz* genes and fluorescent 70 kD dextran were microinjected alone, or with the CTE viral RNA, into the nuclei of NIH 3T3 fibroblasts. Cells were allowed to express the *ftz* RNAs for 20 min then further transcription was inhibited by α-amanitin treatment. After allowing the mRNA to be exported for 2 hours, the cells were fixed and *ftz* mRNA was detected by fluorescence *in situ* hybridization. The distribution of *ftz* mRNA was quantified as described in Figure 3B.

We next experimentally tested whether MSCRs from 5UI^−^ genes promoted mRNA export in tissue culture cells. Indeed, we found that MSCRs from both the *F1 ATP Synthase A* (*F1*) and *ferroredoxin reductase* (*FR*) stimulated efficient nuclear export of the *ftz* transcript (Figure 5A–5B, *F* *1-ftz-Δi*, *FR-ftz-Δi* – see Figure S4 for all modified *ftz* sequences). We note that the alternative export phenotype observed for these MSCRs is at least as robust as any previously observed for SSCR-containing genes. In contrast, we found that the MSCR from the *mitochondrial translation initiation factor 2a* (*MTIF*), a 5UI^+^ gene, does not promote efficient export (Figure 5A–5B, *MTIF-ftz-Δi*). Similar to previous observations with the *MHC* and *Insulin* SSCRs [6], the introduction of seven silent adenine mutations in the *FR* MSCR (*FR7A*) partially inhibited its ability to promote export (Figure 5, *FR7A-ftz-Δi*).

Microinjected mRNA may behave differently from mRNA that has been endogenously transcribed. Therefore, we microinjected plasmids encoding various *ftz* transcripts into the nuclei of NIH 3T3 cells. After allowing the plasmids to be transcribed (20 min), further mRNA synthesis was inhibited by treating cells with the RNA Polymerase II inhibitor α-amanitin. Export of the newly synthesized transcripts was assessed two hours after treatment. We found that transcripts produced from plasmids containing *FR-ftz-Δi*, but not *c-ftz-Δi* or *PTH-ftz-Δi*, were efficiently exported (Figure 5C), as was previously seen for *MHC-ftz-Δi* [6]. Thus, we have shown that MSCR-containing transcripts are capable splicing-independent mRNA export in a manner that depends on 5UI status. This result suggests that the scope of the ALREX pathway extends from ER-trafficked genes to include nuclear mitochondrial genes.

We next wished to assess whether export was dependent on the TAP/p15 nuclear transport receptor, which is required for both SSCR- and splicing-dependent export [6]. We co-injected the viral constitutive transport element (CTE) RNA (known to inhibit TAP/p15 [17]) with the plasmid and observed that export of *in vivo*-transcribed *FR-ftz-Δi* was inhibited. Taken together, these experiments indicate that MSCRs and SSCRs from 5UI^−^ genes promote mRNA export using a similar if not identical pathway.

### Identification of motifs associated with ALREX

Although our experimental findings supported the importance of adenine-depletion for ALREX, they also indicated that other sequence features may be involved. For example, the *PRP* SSCR (from a 5UI^+^ gene) did not promote efficient export even after adenines were eliminated (Figure 3, *PRPΔA-ftz-Δi*). Furthermore, the incorporation of silent adenines only partially inhibited export by the *FR* MSCR (Figure 5, *FR7A-ftz-Δi*), or the *MHC* SSCR [6]. Therefore, we wished to search for additional ALREX-associated sequence features.

Identification of nucleotide motifs responsible for ALREX function is challenging, because enriched RNA-level motifs might arise due to recurrent patterns at the protein sequence level. Although numerous bioinformatics tools exist to search for nucleotide features (such as transcription factor binding sites) in non-coding regions, few are tailored to the problem of identifying RNA motifs within coding regions. We sought to exploit the idea that we have two collections of SSCRs that differ in the expected abundance of ALREX signals. Specifically, we compared SSCRs from genes with and without 5UIs to identify nucleotide signals exhibiting differential abundance between the sets. Although RNA-level features may be artifactually enriched relative to random RNA sequence due to protein sequence-level constraints, such an artifactual enrichment would not be expected in 5UI^−^ relative to 5UI^+^ SSCR-containing genes.

**Figure 6:**
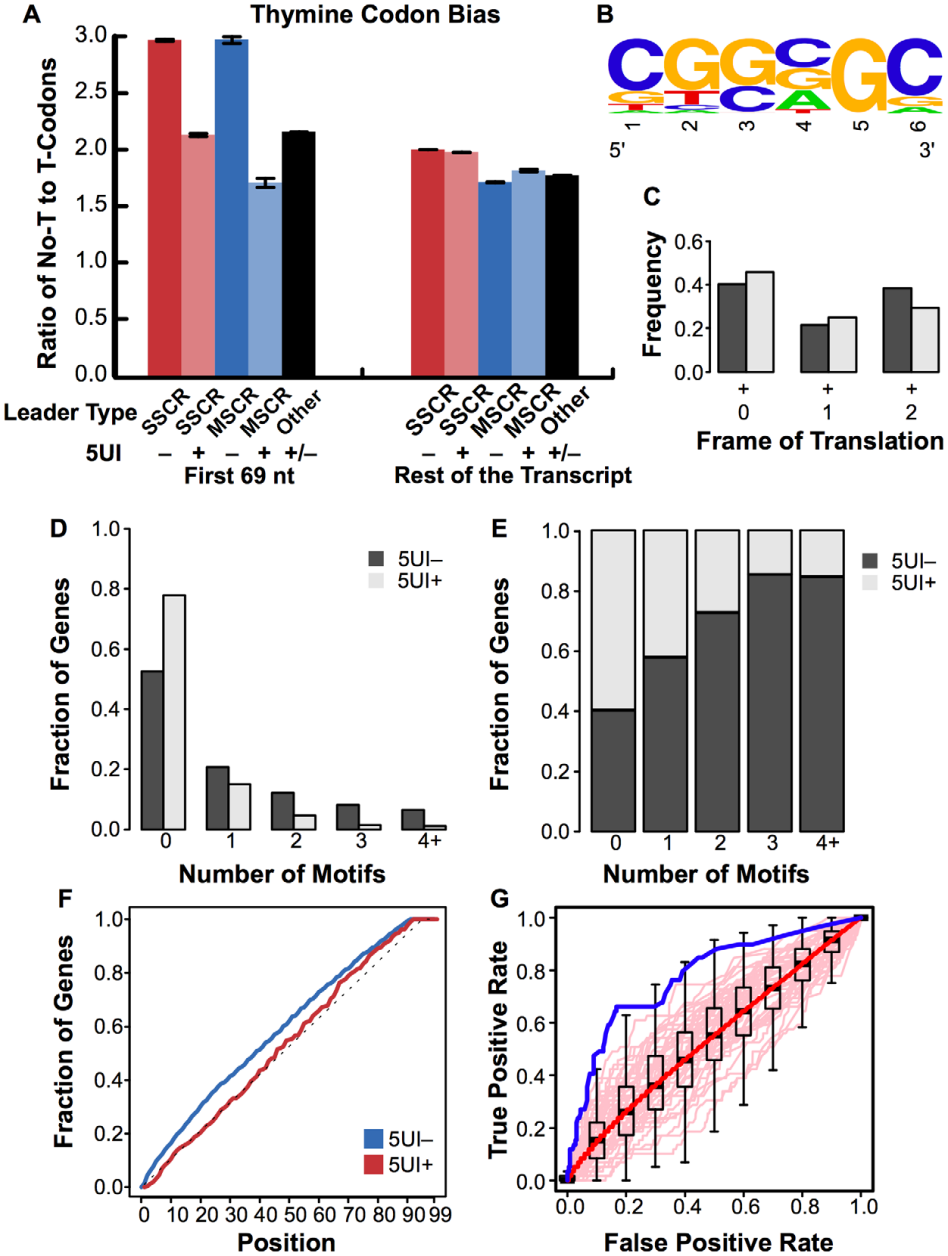
Sequence features associated with ALREX elements. (A) The ratio of thymine-lacking to thymine-containing codons for all amino acids that have both types (A, T, P, H, N, D, R, S, and G) was plotted for the first 69 nucleotides or the rest of the open reading frame of SSCR, MSCR or other genes. As in Figure 2A, all bars represent the average from open reading frames from the human genome which either lacked (“–”) or had (“+”) 5UIs. Error bars represent the standard error of the mean. (B) The position specific scoring matrix corresponding to the 6 nt motif was visualized using WebLogo [40] (C) For each occurrence of the motif, the frame of translation is determined. The fraction of motif occurrences in all three possible frames were plotted for both 5UI^−^ and 5UI^+^ SSCR-containing genes. (D) The distribution of the number of motifs in the set of SSCR-containing genes with 5UIs (negative set) and without 5UIs (positive set) were plotted. (E) For a given number of motif occurrences, the fraction of sequences in the positive versus negative set was plotted. Even though there were ∼2.5 times more sequences in the negative set, the fraction of sequences in the positive set with one or more occurrences of the motif was much higher compared to the fraction in the negative set. (F) The cumulative distribution of the motif occurrences were plotted for both sets (blue line for 5UI^−^ genes and red line for 5UI^+^ genes) and for the uniform distribution (grey line). While the negative set did not differ from uniform distribution, the positive set displayed a left shift towards the 5′ of the transcript. (G) An ROC curve was generated to evaluate the discovered motif's predictive power in identifying the absence of 5UIs among MSCR-containing genes (see Materials and Methods). The performance of the CGSSGC motif is shown with the solid blue line, while the pale pink lines depict the performance of 50 randomly generated motifs. The boxplots represent the interquartile range of TPRs at a specified FPR for all 100,000 random motifs, and whiskers are drawn to 1.5 times the interquartile range. Outliers are not shown, and black horizontal line in each boxplot corresponds to the median TPR at the given FPR. The solid red line is the median performance of all 100,000 random motifs.

We first extended codon usage analyses of the SSCR and MSCR regions to identify other representative signatures. In addition to previously noted adenine depletion, 5UI^−^ SSCR and MSCR genes strongly preferred codons lacking thymine, with a ∼1.4 and a ∼1.7 fold enrichment relative to 5UI^+^ SSCR and MSCR genes (Figure 6A, Fisher's Exact Test *p* = 7×10^-46^ and 4×10^-13^; 95% CI for odds ratio 1.3–1.5 and 1.5–2.0, for SSCRs and MSCRs respectively).

Next, we searched for primary sequence elements using a discriminative motif finding approach. Specifically, we searched for nucleotide sequences that are significantly enriched among SSCR-containing 5UI^−^ genes relative to 5UI^+^ genes using the DEME algorithm [18]. We found a likely candidate motif (Figure 6B), which can be roughly described by the consensus sequence CGSSGC (where S represents a mixture of C and G). This motif is highly depleted of adenines and thymines consistent with our analysis (Figure 2, Figure 3, and Figure 6A) and had high information content.

The motif did not show a strong preference for a particular frame of translation (Figure 6B) suggesting that this signal is relevant at the RNA as opposed to protein level. The motif not only appeared in a higher fraction of 5UI^−^ SSCR sequences (47.5% versus 22.2% in 5UI^+^ SSCRs; see Materials and Methods), but also was much more likely to occur in multiple copies in the SSCRs of 5UI^−^ genes (Figure 6C, 6D; 26.8% versus 7.14%). The CGSSGC motif also revealed a strong positional bias, occurring more frequently toward the 5′ end of coding regions from 5UI^−^ genes (Figure 6E, Figure S5, Wilcoxon Rank Sum Test p = 0.002, median position was 39 and 45 among 5UI^−^ and 5UI^+^ genes, respectively; see Materials and Methods).

We wished to further examine the question of whether the non-canonical mRNA export function of SSCRs is acting via the same mechanism as that of MSCRs. We therefore tested whether the CGSSGC motif (which was enriched among 5UI^−^ SSCR genes) could also predict the absence of 5UIs among genes with an MSCR. We compared performance of the CGSSGC motif (discovered without use of any MSCR-containing genes) in discriminating 5UI^−^ from 5UI^+^ MSCRs and found it to outperform at least 99% of 100,000 randomly generated motifs (Figure 6F; False-positive Rate range 10% to 70%; see Materials and Methods). This result indicates that MSCRs and SSCRs, despite differences in the protein sequences they encoded, each play host to a common RNA-level motif associated both with the lack of 5UIs and the ability to support non-canonical mRNA export.

To identify additional motifs, we used the AlignACE [19] algorithm on the set of SSCR sequences from 5UI^−^ genes. This algorithm has the advantage that it can identify multiple nucleotide sequences and allows greater flexibility in motif length. We filtered the discovered sequences for their discriminative ability and found 19 motifs that were significantly enriched among 5UI^−^ relative to 5UI^+^ genes (Table S2, see Materials and Methods). The discovered motifs displayed mutual similarity and included several close variants of the CGSSGC motif discovered by DEME (See Figure S6 for the PSSM logos of the most discriminative AlignACE motifs).

We next focused on the properties of the four most discriminative AlignACE motifs. All four motifs were more likely to occur in multiple copies among the 5UI^−^ genes compared to 5UI^+^ genes (Figure S7). Even though these four motifs were discovered based on their ability to discriminate 5UI^−^ from 5UI^+^ genes among those genes with SSCRs, these motifs were also predictive of 5UI absence for genes with MSCRs (Figure S8). All four motifs performed in the top quartile compared to 100,000 random motifs (Figure S8; see Materials and Methods). However, unlike the CGSSGC motif, three of these motifs displayed a significant bias for occurring in a particular frame of translation. These three motifs may thus be detecting protein sequence-level differences between 5UI^−^ and 5UI^+^ genes (Figure S9). In fact, consensus sequences of many AlignACE motifs included CTGs that can encode leucines, which were highly enriched among SSCRs and MSCRs from 5UI^−^ genes relative to their 5UI^+^ counterparts.

**Figure 7:**
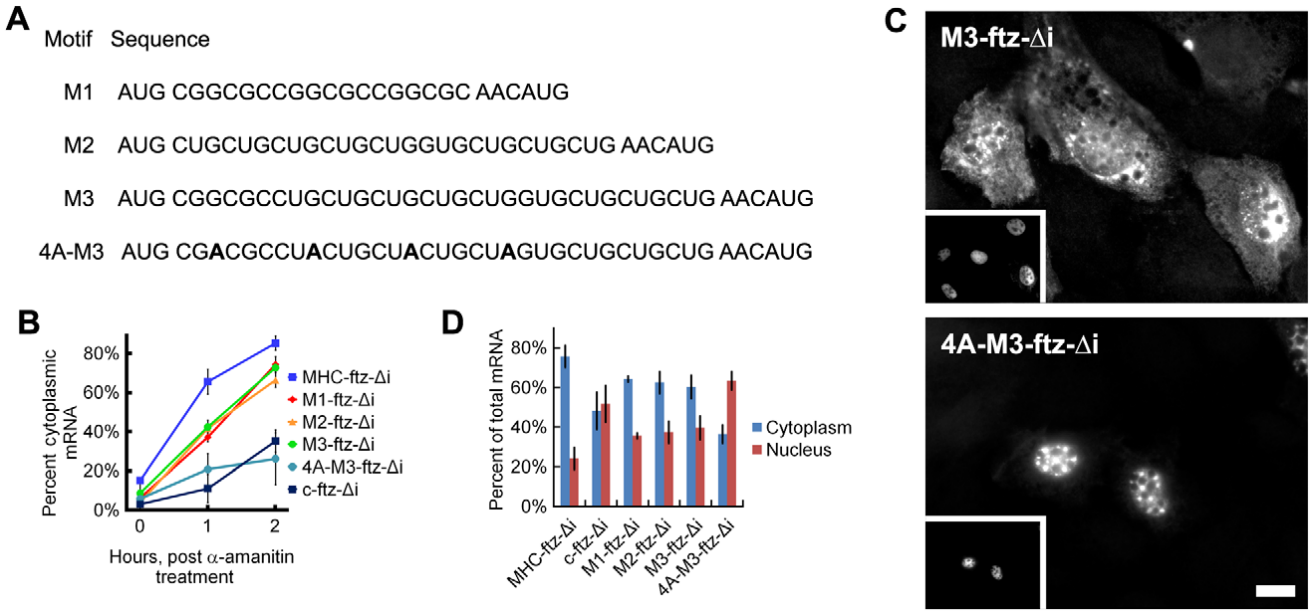
ALREX–associated motifs promote nuclear export of mRNA. Various RNA motifs were inserted just after the start codon of the *c-ftz-Δi* construct. The nucleotide sequences of each RNA insert are depicted in (A). Note that the 4 silent adenine mutations used to convert *M3* into *4A-M3* are indicated in bold. (B) DNA plasmids containing various versions of *ftz* were microinjected along with fluorescent 70 kD dextran into the nuclei of NIH 3T3 fibroblasts. After 20min, α-amanitin was added to halt further transcription and the cells were allowed to export the RNA for the indicated time periods. Cells were fixed, stained for *ftz* mRNA and export was quantified as in Figure 3B. Each bar represents an average of three separate experiments, each of which consisted of 15–30 cells. Error bars represent the standard deviation of the three experiments. (C) Representative cells from (B) microinjected with DNA plasmids containing either *M3-ftz-Δi* or *4A-M3-ftz-Δi* and fixed 2 hours after α-amanitin treatment. Cells were imaged for *ftz* mRNA by FISH and fluorescent 70 kD dextran (insets). Scale Bar = 15 µm. (D) COS-7 cells were transfected with plasmids containing various versions of *ftz* and allowed to express the mRNA overnight. The cells were then fixed and stained for *ftz* mRNA by FISH and the cytoplasmic over the total fluorescence signal was quantified as in Figure 3B. Each bar represents an average of five separate experiments, each of which consisted of 20–50 cells. Error bars represent the standard error of the mean between the experiments.

We next decided to test whether synthetic elements matching the discovered motifs could promote the export of *ftz* mRNA. We used versions of the *ftz-Δi* mRNA containing either three copies of an element matching the consensus CGSSGC motif (*M1-ftz-Δi*), a CUG repeat-containing element (*M2-ftz-Δi*), or a single copy of each (*M3-ftz-Δi* see Figure 7A for the sequences of all these constructs). We chose CUG repeats as they appeared in many of the consensus sequences of AlignACE motifs (Table S2). In addition, there are several RNA binding proteins, such as CUG-BP1 [20] and the Muscleblind family of proteins [21] that are known to recognize CUG repeats.

To assay for export activity we microinjected plasmids that contained versions of the *ftz* gene fused to segments containing elements matching ALREX-enriched motifs and their combinations (Figure 7A) into the nuclei of NIH 3T3 cells. After allowing the plasmids to be transcribed (20 min), further mRNA synthesis was inhibited by treating cells with α-amanitin. We found that all three motif-containing *ftz* constructs (*M1-, M2-, M3-ftz-Δi*) were exported more efficiently than *c-ftz-Δi* but substantially less efficiently than *MHC-ftz-Δi* mRNA (Figure 7B). Adenine depletion was required for export, as mRNA generated from plasmid containing a mutant form of *M3-ftz-Δi* bearing four silent adenine mutations (*4A-M3-ftz-Δi*, see Figure 7A) collectively disrupting each of the two component elements was not efficiently exported (Figure 7B–7C). To further validate these results, we transfected plasmids encoding the motif-containing *ftz* genes with elements corresponding to these motifs into COS-7 cells and measured the steady state distribution of mRNA. In agreement with our microinjection experiments, we found that the three motif-containing *ftz* constructs were exported to a level that was clearly higher than *c-ftz-Δi* but lower than *MHC-ftz-Δi* (Figure 7D). As observed for microinjected NIH3T3 cells (Figure 5), mRNA generated from a plasmid containing the *4A-M3-ftz-Δi* construct was not efficiently exported from transfected COS-7 cells (Figure 7D).

## Discussion

The function and evolution of introns has been intensely studied since their discovery (reviewed in [22], [23]). Despite the presence of a large number of introns in untranslated regions, especially in the 5′ untranslated regions of transcripts, these studies have been largely focused on introns in coding regions [3]. We established that the distribution of 5UIs in the human genome is non-random, with specific functionally related groups of genes being enriched [1] or depleted (this study) for 5UIs. Here we show that, in both secreted and mitochondrial genes, the presence or absence of 5UIs correlates with sequence features at the beginning of the coding region. Minimally, our results further support the conclusion that complex selective forces govern the evolution of 5′UTR introns. Moreover, our results are best explained by the existence of a regulatory mechanism that is both special to 5UIs and has relevance to thousands of genes across the genome.

**Figure 8:**
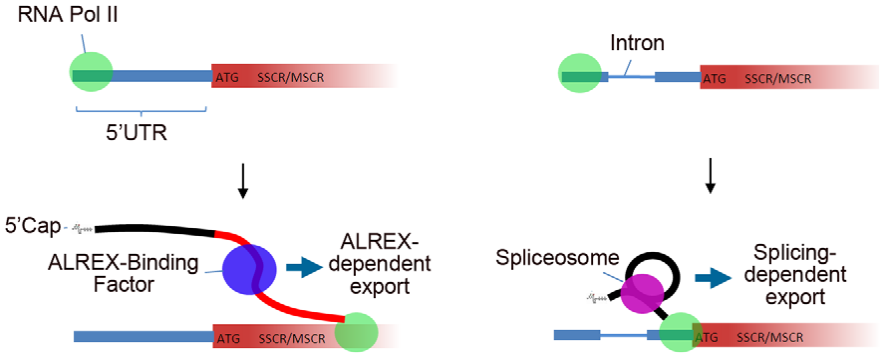
Model describing 5′UTR intron effects on nuclear mRNA export by genes with SSCR/MSCRs. (A) In the absence of 5UIs, the SSCR/MSCR is transcribed prior to any splicing event and as a consequence the transcript is exported via the ALREX pathway. (B) The presence of the 5UI results in recruitment of the splicesome and associated accessory proteins before the SSCR/MSCR is transcribed and hence the transcript is exported by the splicing-dependent pathway. As this transcript is not exported by the ALREX pathway, there is no selection pressure for the SSCR/MSCR to maintain specific nucleotide features associated with ALREX.

Our results show that nuclear transcripts encoding both secretory and mitochondrial proteins share RNA-level signals capable of directing mRNA export, even for an intronless message. It has frequently been observed that mRNAs of functionally related genes are co-regulated at the post-transcriptional level (‘the regulon hypothesis’ [24]). Our results suggest that, consistent with this phenomenon, the ALREX pathway can facilitate coordinated expression of functionally related genes at the level of mRNA export. Moreover, our analyses support a model whereby the first transcript element emerging from RNA Polymerase II during transcription—be it an intron or an ALREX-promoting element—determines which RNA export pathway is predominantly followed (Figure 8). Under this model, presence of a 5UI would supersede downstream SSCR or MSCR export signals and relax selection pressures that maintain ALREX-promoting sequence features.

Although we have made progress in defining some sequence features that mediate the ALREX function (see Figure 6 and Figure 7), it is clear that a more extensive description of ALREX features individually and in combination will be quite useful. We found specific nucleotide-level motifs in the 5′ end of coding regions which discriminate between genes with and without 5UIs. Substantial future efforts will be required to combine information about 5UI absence with the presence and placement of ALREX signals within a unified framework that can predict ALREX activity. This information could be used to compile a full list of transcripts using the ALREX pathway. It will be interesting to determine whether other genes, such as those that encode membrane-bound proteins but lack a signal sequence (and hence an SSCR), can use this alternative export pathway.

The most fundamental challenge for future studies will be to understand the biological role or roles of the ALREX pathway. Why is its selection maintained even in transcripts that contain coding region introns and are therefore enabled to use the canonical mRNA export pathway?

Although the functional downstream consequences of using either the splicing-dependent or ALREX-pathway remain unknown, silent mutations within the SSCR not only impair mRNA export but also disrupt proper ER-targeting of the transcripts [6]. This suggests that multiple post-transcriptional events, such as mRNA export, mRNA transport in the cytoplasm and mRNA translation, are coupled [25].

Here, we have discovered and validated two motifs that promote mRNA export, suggesting that ALREX may recruit more than one nuclear factor. Such factors could not only dictate RNA export but perhaps also dictate how the mRNA is distributed and translated once in the cytoplasm. Investigation of these questions awaits identification of ALREX factors, and of mRNA localization or other phenotypes associated with disrupted ALREX function. One of the motifs we discovered is a long CUG repeat that could potentially bind to CUG binding proteins. However, *MHC-ftz-Δi* mRNA is exported from HeLa cells that were depleted of both *MBNL1* and *MBNL2*, two members of the muscleblind family of CUG-repeat binding proteins (unpublished findings), suggesting that these are not the responsible factors. Identification of the ALREX-element binding protein(s) will shed light onto how ALREX operates and provide insight into the biological role of this pathway.

The question of biological role is particularly intriguing in the case of nuclear-encoded mitochondrial genes. The textbook description of nuclear-encoded mitochondrial genes has translation of these genes occurring within the general pool of cytoplasmic proteins, with subsequent protein localization due solely to the mitochondrial targeting peptide sequence. However, there is evidence that nuclear-encoded mitochondrial transcripts can localize to the vicinity of mitochondria prior to translation [26], [27]. Although we do not detect any mitochondrial targeting of MSCR-bearing transcripts (Figure 5), it is possible that a fraction of these mRNAs are indeed localized. It will be interesting to learn what role ALREX could play in the localization and translation of nuclear-encoded mitochondrial genes.

Substantial future studies will be required to further explore mechanisms of the ALREX pathway. For example, it is unclear whether ALREX signals are inhibited by other complexes deposited on the transcript in a splicing dependent manner. One example is the Exon Junction Complex (EJC), which potentiates the translation of properly spliced mRNA [28], [29] and the nonsense-mediated degradation of improperly spliced transcripts [30], [31]. Some mRNAs, such as those of *PrP* and *PTH* genes, encode secreted proteins but lack any ALREX-promoting element. For such mRNAs, it is possible that the proper ER- targeting and efficient translation of these transcripts requires the recruitment of the EJC or TREX components to the 5′UTR. Identification of the nuclear proteins that associate with ALREX elements, and how these factors are coupled to other processes, will yield significant insight into the role of ALREX in mediating gene expression, and localization of both mRNAs and proteins.

## Materials and Methods

### Assembly of a collection of genes with 5UIs and analysis of total exonic and intronic length

NCBI's human Reference Gene Collection (RefSeq) [32] and the associated annotation table, retrieved from the UCSC genome browser genome assembly May 2004 (http://hgdownload.cse.ucsc.edu/downloads.html), were used to extract a high confidence set of 5UIs. The lengths of 5′UTR-associated genomic features were determined using RefSeq intron-exon definitions (downloaded June 2007). Out of a total ∼24.5 k RefSeq transcripts, ∼8.5 k contained at least one intron. Genomic coordinates of 5UIs examined were as previously described [1]. When multiple splice variants involving a given 5′UTR exhibited identical splicing patterns within that 5′UTR region, a single identifier was selected randomly as the representative for that 5′UTR.

For the remaining transcripts, total lengths of coding region introns were determined from the RefSeq Annotation (downloaded from UCSC genome browser, May 2004 genome assembly on May 15^th^ 2009 http://hgdownload.cse.ucsc.edu/downloads.html).

### Microinjection and mRNA imaging

DNA constructs encoding *ftz* isoforms were assembled by first digesting the pBR322 plasmid containing *c-ftz-Δi* [6] with Nco I and ligating oligonucleotides encoding various SSCRs and MSCRs (see Figure S4) so that the extra sequences were all inserted just downstream of the start codon. The constructs were then transcribed into mRNA, which was then polyadenylated, purified and then microinjected into NIH 3T3 fibroblast nuclei at 200 µg/ml with Alexa488 conjugated 70 kD dextran (1 mg/ml) as previously described [6], [33]. DNA microinjections were performed as previously described [6]. Briefly, *ftz* isoforms were subcloned into pCDNA3 using Hind III and Xho I and microinjected at 50 µg/ml with Alexa488-conjugated 70 kD dextran (1 mg/ml) into NIH 3T3 fibroblast nuclei. After allowing the RNA to be transcribed for 20 min, the cells were treated with α-amanitin (50 ng/ml) to prevent further transcription. CTE RNA was synthesized as previously described [6] and microinjected at a concentration of 200 µg/ml along with DNA and Alexa488-conjugated 70 kD dextran. All microinjected cells were incubated for the indicated time to allow for mRNA export, then fixed with 4% paraformaldehyde in phosphate buffered saline (PBS). DNA transfections into COS-7 cells were performed as described previously [6]. Transfected cells were incubated for 12–18 hrs, then fixed with 4% paraformaldehyde in PBS. The *ftz* mRNA was stained by fluorescence *in situ* hybridization followed by imaging and quantification of RNA nuclear export as previously described [6]. Cell imaging and mRNA quantification were also performed as previously described [33].

### Functional enrichment of Gene Ontology categories

FuncAssociate [34], [35] beta version was used for Gene Ontology (GO) analysis, and Synergizer [36] was used for mapping RefSeq IDs into the ‘namespace’ of GO association files using Ensembl as the synonym authority. We restricted the space of genes in which GO correlations were sought to RefSeq because our 5UI genes were drawn only from this set. To quantify the effect size of GO correlations, the results in Table S1 were sorted according to their log_10_ odds ratio, with significance calculated by Fisher's Exact Test as previously described [35]. Multiple hypothesis correction was achieved via a resampling approach that preserves the dependency structure between the tested hypotheses [35]. Adjusted *p*-values were calculated using 10000 resampling simulations.

### Analysis of SSCR and MSCR sequences

We retrieved the complete set of transcripts with signal peptide annotations from the Ensembl 50 database using Biomart [37] (downloaded on February 2009 http://www.ensembl.org/biomart/martview). Of the 38396 transcripts in this database, 4953 were annotated as having a signal peptide, and 4704 of these were in our set of RefSeq genes. The coding region sequences for all the genes in our set were downloaded from NCBI Refseq Collection release 33 (ftp://ftp.ncbi.nih.gov/refseq/H_sapiens/mRNA_Prot). The ratio of the amino acids, total adenine counts and the codon usage bias were calculated for the first 69 nt and the rest of the sequences. There were 135 coding region sequences that had a length that was not a multiple of three. These sequences in addition to those with total length less than 150 nt were removed from further analysis.

The list of mitochondrial genes was retrieved from the Organelle DB [38] website (downloaded on February 2009 from http://organelledb.lsi.umich.edu/). Identifiers were translated to RefSeq ID using Synergizer [36]. Nine genes were removed from this list as they were encoded by the mitochondrial genome. For some genes, Synergizer could not find a RefSeq ID corresponding to the “standard name”. These genes were manually inspected and the synonyms provided by Organelle DB website were used to find corresponding RefSeq IDs. When multiple splice variants were exact duplicates with respect to the first 69 nts of their coding region, a single identifier was selected as the representative. This procedure yielded 364 RefSeq transcripts out of ∼25 k transcripts having an MSCR. The manually edited list of mitochondrial genes is available in Dataset S1. The software package R 2.6.0 was used for all the statistical analyses, except where otherwise noted.

### Motif discovery, scoring, and disambiguation of overlaps

For motif discovery, the first 99 nt of SSCR-containing genes were used to ensure that all signal peptides were included in their entirety. Highly similar sequences were removed to avoid overweighting closely related sequences. Specifically, the first 99 nt from each sequence was aligned to all others using blastn [39]. A threshold (*E*-value <10^−25^) was used to group similar sequences, and one randomly selected representative from each such set was used after this filter.

We used the DEME [18] software to search for a motif that is highly enriched in the 5UI^−^ set of sequences relative to the 5UI^+^ set. We also used the AlignACE software [19] to search for a set of highly enriched motifs in the 5UI^−^ set. AlignACE searches for frequently occurring motifs in both the forward and complementary strands of DNA sequences. Choosing to focus on RNA motifs, we discarded 2 of the 20 motifs reported that were constructed from less than 10 representative forward-strand sites.

Positional Specific Scoring Matrices (PSSM) of the discovered motifs were extracted from the forward-strand sites of each motif. For a given sequence *s* and a motif with length *m*, all windows of size *m* within the first 99 bases were scored using the PSSM of the motif. To avoid calling multiple overlapping motifs, only the highest scoring window in a contiguous series of overlapping windows was selected. For each motif, an initial PSSM score threshold (t^*^) was selected such that t^*^ yields the highest enrichment of motif-containing sequences among the SSCR-containing and 5UI^−^ genes on the p-value generated from Fisher's Exact Test using the 2×2 contingency table (Table 1).

**Table 1:** 2×2 Contingency Table to determine PSSM threshold t*.

# of SSCR-containing and 5UI^−^ genes matching the motif according to t*	# of SSCR-containing and 5UI^+^ genes not matching the motif according to t^*^
# of SSCR-containing and 5UI^−^ genes not matching the motif according to t^*^	# of SSCR-containing and 5UI^+^ genes not matching the motif according to t^*^

Given the total number of genes *N*, the number of 5UI^−^ genes *m*, and the number of motif-containing sequences *n*, this test estimates the probability that *k* or more genes would be found to overlap between the 5UI^−^ genes and the motif-containing sequences under the null hypothesis of independence:
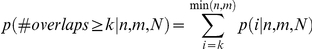
where the probability of observing exactly *i* overlaps given *N*, *m* and *n* follows from the hypergeometric distribution:
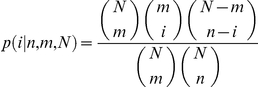



Among the 18 AlignACE motifs, we focused on the four that were most enriched among 5UI^−^ genes compared to 5UI^+^ genes based on the resulting *p*-value. Further analyses on motif occurrences and positional distributions were performed on these four AlignACE motifs and the DEME motif.

### PSSM score threshold selection

While PSSM threshold selection using Fisher's Exact Test provided a quick way identify discriminative AlignACE motifs, the selection of thresholds did not take into account the likelihood that such discrimination may have occurred by chance.

To account for this possibility, we randomly generated four sets of PSSMs matching the discovered motifs' lengths (6, 10, 14, and 16 nt). We modeled each position of the PSSM as an independent sample from a Dirichlet distribution with parameters (α_i_) equal to the background nucleotide frequency such that Σα_i_ = 1. The background nucleotide frequency was calculated among the first 99 nts of either SSCR-containing or MSCR-containing 5UI^−^ genes. For each given motif length, we generated 40,000 random PSSMs for SSCR set and 100,000 random PSSMs for the MSCR set. We generated receiver operating characteristic (ROC) plots to compare the discriminative performance of these randomly generated PSSMs with that of the discovered motifs. First, we scanned each sequence to find the maximum score for each PSSM. We classified a sequence as motif-containing if its maximum PSSM score was greater than a given threshold t*. For all random and discovered motifs, we calculated the true positive rate (TPR) as the fraction of motif-containing 5UI^−^ genes, and the false positive rate (FPR) as the fraction of motif-containing 5UI^+^ genes as a function t*. Therefore, each point on an ROC plot corresponds to (TPR, FPR) of a particular PSSM at some threshold t*. These ROC plots are informative about the analyzed motif's power to discriminate 5UI^−^ from 5UI^+^ genes.

For each discovered motif, we used the ROC plots generated from SSCR-containing genes (Figure S8) to choose the PSSM score threshold value (t′) for subsequent analysis. The threshold t′ was chosen such that it maximizes the difference between the discovered motif's TPR and the median TPR of the random motifs was the most at the FPR value corresponding to t′. Since we discovered motifs using the SSCR-containing set only, the ROC plots for the MSCR set were not subject to any overfitting that might have occurred during motif discovery.

### Positional distribution of sequence elements that match the motifs

To assess whether there is any significant deviation in the positional distribution of motifs in the 5UI^−^ set from that in the 5UI^+^ set, we performed the Wilcoxon Rank Sum test. We examined differences in distributions for the positions of all motif occurrences in each sequence. We also generated histograms for the reading frame at which motifs occur in the coding region to look for differences between the 5UI^−^ and 5UI^+^ sets.

## Supporting Information

Dataset S1 List of RefSeq Identifiers of Nuclear-Encoded Mitochondrial Genes.
(0.04 MB XLS)

Figure S1 The depletion in 5′UTR introns is not attributable to differences in 5′UTR length. (A) 5′UTR length was calculated as the cumulative length of all 5′UTR exons. Boxplot showing the differences between the distributions of lengths of 5′UTRs from 5UI^+^ or 5UI^−^ genes was drawn as in Figure S2. (B) Differences in 5′UTR length between SSCR ‘+’, MSCR ‘+’, and SSCR ‘–’ genes was shown using a boxplot. Genes with SSCRs and MSCRs have significantly shorter 5′UTRs. (C) A histogram of log_10_ of total 5′UTR length and the fitted kernel density estimate was plotted for 5UI containing genes. (D) A histogram of log_10_ of total 5′UTR length and the fitted kernel density estimate was plotted for 5UI^−^ genes.
(0.07 MB PDF)

Figure S2 SSCR-containing genes do not differ from other genes with respect to total length of non-5′UTR introns. (A) The 25^th^ to 75^th^ quartile in log_10_ of total length of non-5′UTR introns was represented with a boxplot for both SSCR-containing (+), and -lacking (–) genes. Whiskers were drawn to 1.5 times the inter-quartile range. No statistically significant differences were observed. (B) Histogram of log_10_ of total length of non-5UIs for SSCR containing genes and the fitted normal distribution is plotted. The distribution of the non-5UI lengths of these genes does not differ from the normal distribution with a mean of ∼4.2 and a standard deviation of ∼0.7 (Kolmogorov-Smirnov test *p*-value = 0.8).
(0.22 MB PDF)

Figure S3 Synonymous codon bias against adenines in SSCRs and MSCRs derived from genes lacking 5′UTR introns. (A) The ratio of adenine-lacking to adenine-containing codons was plotted for the first 69 nucleotides or the rest of the open reading frame from genes. Sequences were divided into separate groups based on the leader sequence type and 5UI presence/absence. Bars represent the mean ratio, and the standard error of the mean was shown. (B) The ratio of adenine-lacking to adenine-containing codons was plotted as in panel (A).
(0.03 MB PDF)

Figure S4 Nucleotide sequences of experimentally tested SSCRs and MSCRs. Mutations in the *PRPΔA* and *FR7A* sequences are indicated in bold.
(0.08 MB DOCX)

Figure S5 The CGSSGC motif tends to be positioned near the 5′ end among 5UI^−^ genes. The histograms represent the position of all occurrences of the CGSSGC motif. The black line corresponds to fraction of motifs positions among 5UI^−^ genes while the grey line corresponds to that among 5UI^+^ genes.
(0.03 MB PDF)

Figure S6 Representation of motifs enriched among SSCR-containing 5UI^−^ genes. WebLogo server [40] was used to visualize the position specific scoring matrices corresponding to eight AlignACE motifs that were most enriched among 5UI^−^ genes. Letter height within each logo reflects the frequency of nucleotides at each position. The panels are in descending order from most enriched motif (panel A) to least enriched motif (panel H) among 5UI^−^ genes.
(0.09 MB PDF)

Figure S7 Fraction of genes with one or multiple copies of the discriminative motifs discovered by AlignACE. AlignACE motifs that were most enriched among 5UI^−^ genes are shown in descending order of enrichment (panel A-D). The left panels show the distribution of the number of motifs in the set of SSCR-containing genes with 5UIs (negative set) or without 5UIs (positive set). The right panels show the fraction of sequences in the positive versus negative set for a given number of motif occurrences. For all four motifs shown, the positive set was enriched for the motif, both in terms of fraction of sequences with at least one copy of the motif [(A) 54.1% versus 28.5%; (B) 57.7% versus 36.8%; (C) 59.1% versus 38.5%; and (D) 61.5% versus 41.2%] and in terms of fraction of sequences with multiple motif occurrences [(A) 32.4% versus 11.8%; (B) 28.0% versus 13.2%; (C) 31.0% versus 12.8%; and (D) 34.5% versus 17.9%].
(0.03 MB PDF)

Figure S8 Discriminative motifs discovered by AlignACE are also predictive of 5UI absence among MSCR-containing genes. ROC plots are as described in Figure 6G for the four AlignACE motifs that were most enriched among 5UI^−^ genes in descending order of enrichment (panel A-D).
(1.06 MB PDF)

Figure S9 Three of the four discriminative motifs discovered by AlignACE reveal a strong bias for a particular frame of translation. The four AlignACE motifs that were most enriched among 5UI^−^ genes, in descending order of enrichment (panel A-D). The positions of all motif occurrences were classified into one of three possible frames of translation. The fraction of motif occurrences in each frame of translation was plotted for both 5UI^−^ and 5UI^+^ genes.
(0.03 MB PDF)

Table S1 5UIs are depleted from ER-Targeted and Mitochondrial Genes. The GO analysis was done using the FuncAssociate [34], [35]. The functional categories are sorted in descending order based on their log-odds score (LOD). All categories with a LOD score less than -0.1 and *p-value*< 0.05 are reported. Fisher's Exact Test was used to calculate the statistical significance and correction for multiple hypothesis testing was done using a resampling approach (see [35] for details).
(0.07 MB PDF)

Table S2 19 motifs discovered by AlignACE are significantly represented among 5UI^−^ Genes. AlignACE motifs that were enriched among 5UI^−^ genes and the DEME motif were shown. There were 938 and 2594 total sequences in the 5UI^+^ set and the 5UI^−^ set, respectively. Fisher's exact test was used to test the significance of enrichment of motif containing genes among 5UI^−^ genes.
(0.04 MB PDF)

Text S1 The depletion of 5UIs from SSCRs and MSCRs is not attributable to differences in 5′UTR length.
(0.15 MB PDF)
