# Supplementary figures and images for: Genome Analysis Reveals Interplay between 5′UTR Introns and Nuclear mRNA Export for Secretory and Mitochondrial Genes
Source: PLoS Genet. 2011 Apr 14;7(4):e1001366. doi: 10.1371/journal.pgen.1001366 (PMC3077370; doi:10.1371/journal.pgen.1001366)

Figure S1

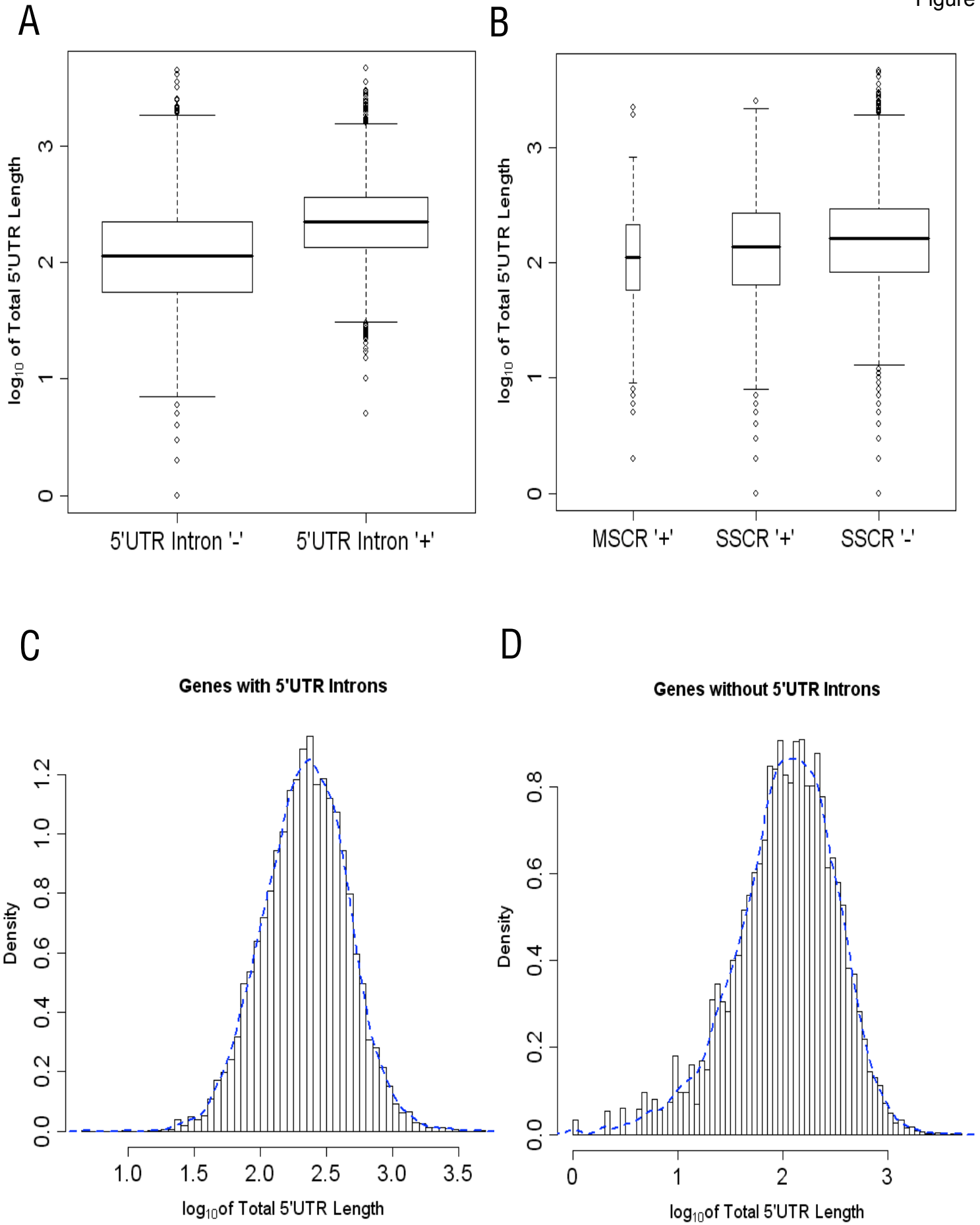

Supplement: Figure S1 — The depletion in 5′UTR introns is not attributable to differences in 5′UTR length. (A) 5′UTR length was calculated as the cumulative length of all 5′UTR exons. Boxplot showing the differences between the distributions of lengths of 5′UTRs from 5UI+ or 5UI− genes was drawn as in Figure S2. (B) Differences in 5′UTR length between SSCR ‘+’, MSCR ‘+’, and SSCR ‘–’ genes was shown using a boxplot. Genes with SSCRs and MSCRs have significantly shorter 5′UTRs. (C) A histogram of log10 of total 5′UTR length and the fitted kernel density estimate was plotted for 5UI containing genes. (D) A histogram of log10 of total 5′UTR length and the fitted kernel density estimate was plotted for 5UI− genes. (0.07 MB PDF) [file pgen.1001366.s002.pdf]

Figure S2

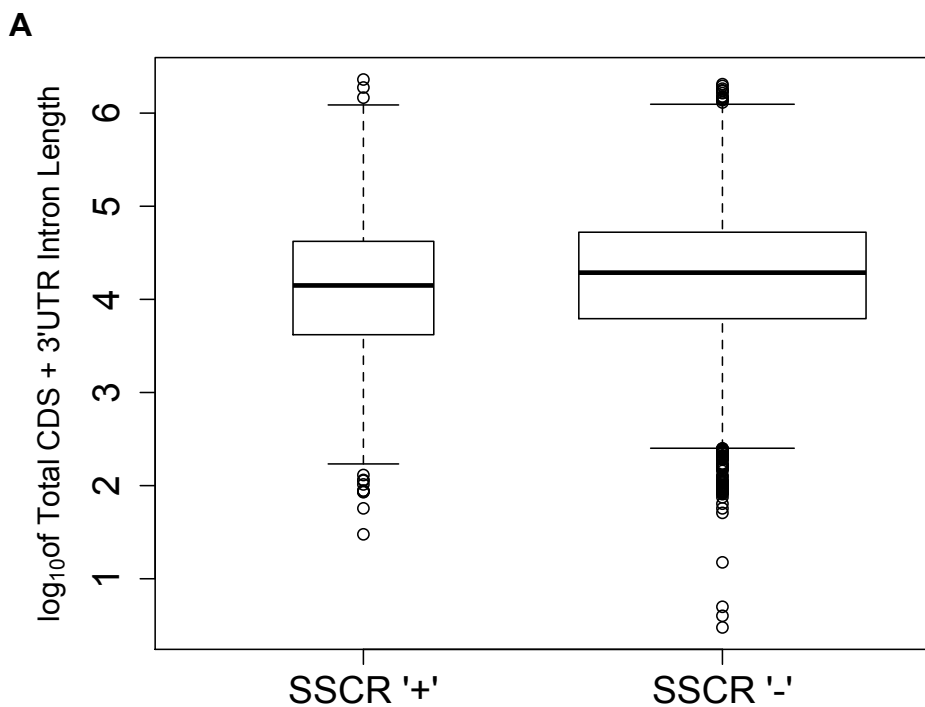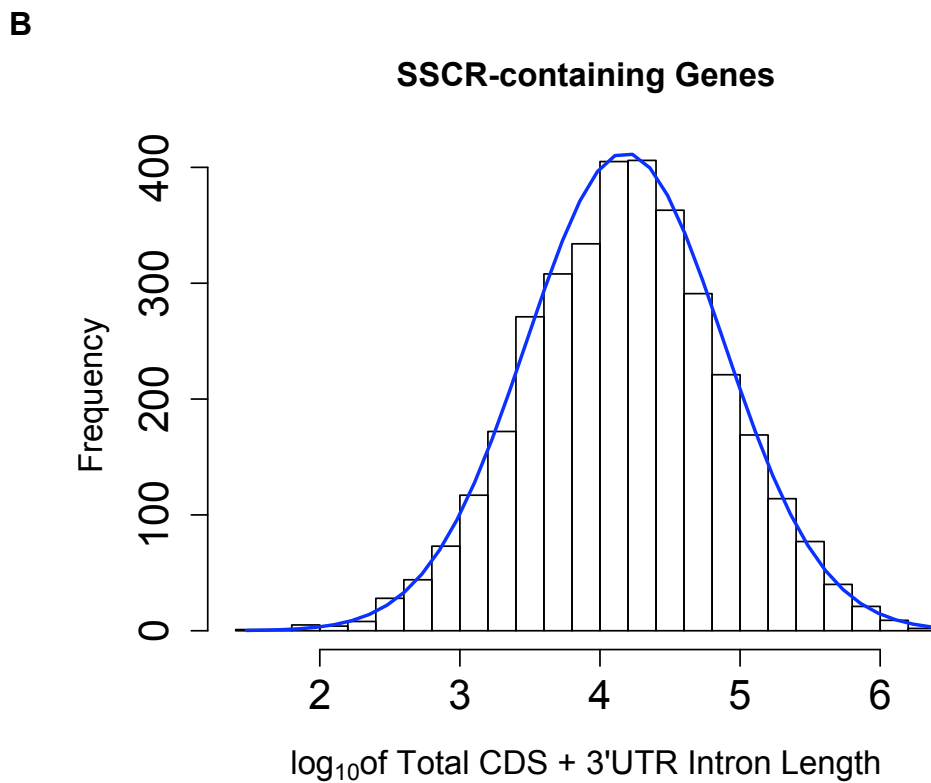

Supplement: Figure S2 — SSCR-containing genes do not differ from other genes with respect to total length of non-5′UTR introns. (A) The 25th to 75th quartile in log10 of total length of non-5′UTR introns was represented with a boxplot for both SSCR-containing (+), and -lacking (–) genes. Whiskers were drawn to 1.5 times the inter-quartile range. No statistically significant differences were observed. (B) Histogram of log10 of total length of non-5UIs for SSCR containing genes and the fitted normal distribution is plotted. The distribution of the non-5UI lengths of these genes does not differ from the normal distribution with a mean of ∼4.2 and a standard deviation of ∼0.7 (Kolmogorov-Smirnov test p-value = 0.8). (0.22 MB PDF) [file pgen.1001366.s003.pdf]

## Leucine Codon Bias

A

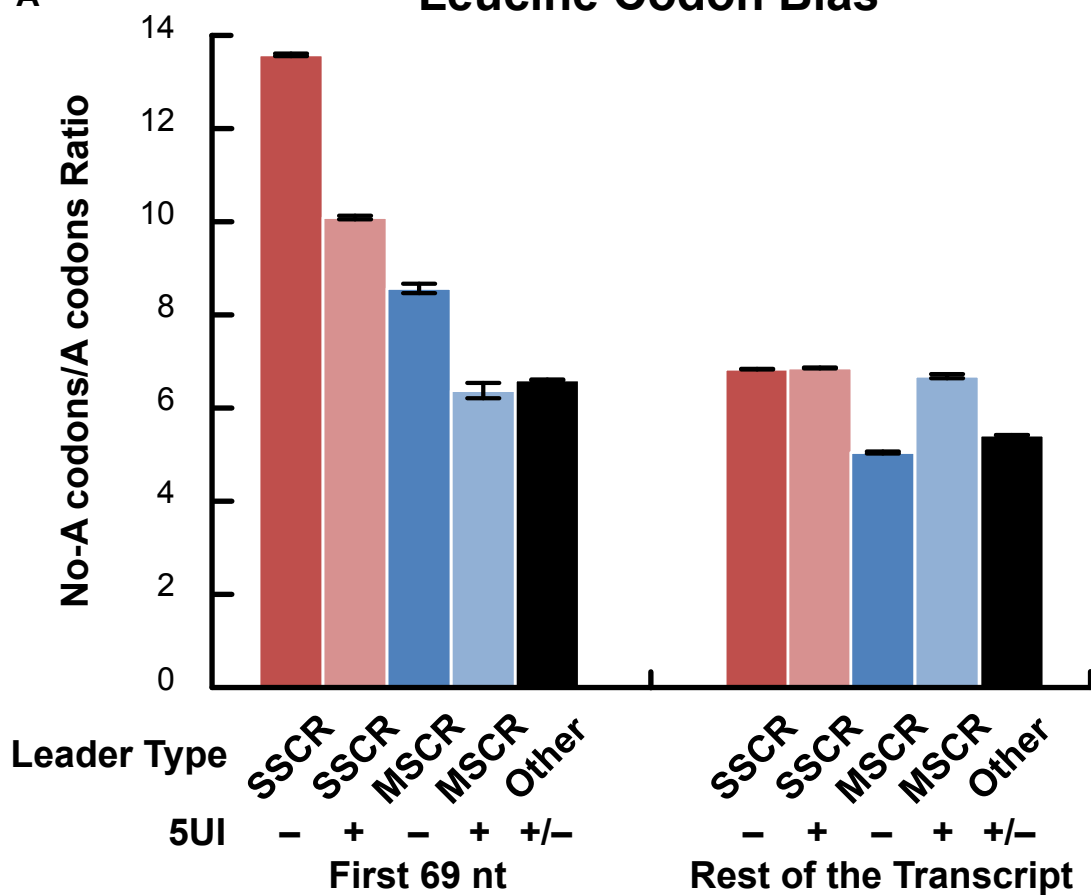

## Serine Codon Bias

B

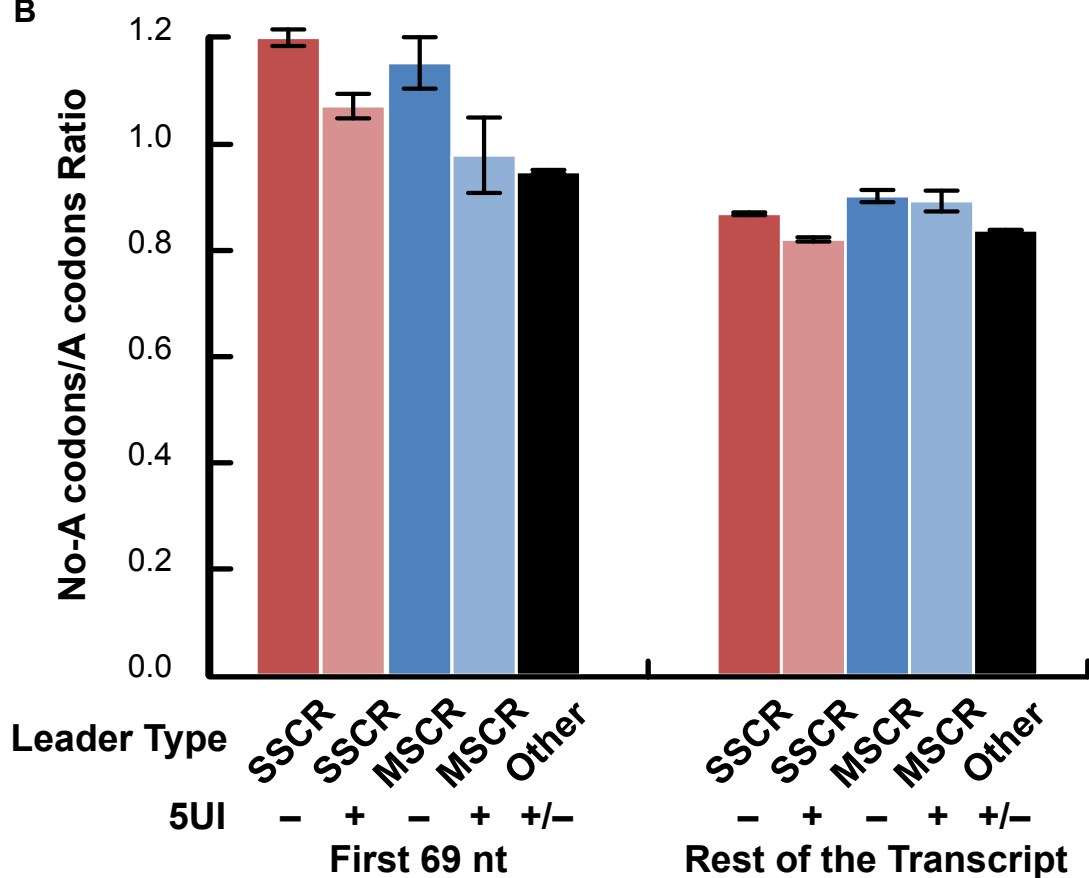

Supplement: Figure S3 — Synonymous codon bias against adenines in SSCRs and MSCRs derived from genes lacking 5′UTR introns. (A) The ratio of adenine-lacking to adenine-containing codons was plotted for the first 69 nucleotides or the rest of the open reading frame from genes. Sequences were divided into separate groups based on the leader sequence type and 5UI presence/absence. Bars represent the mean ratio, and the standard error of the mean was shown. (B) The ratio of adenine-lacking to adenine-containing codons was plotted as in panel (A). (0.03 MB PDF) [file pgen.1001366.s004.pdf]

Figure S5

### Positions of All Occurrences of the Motif

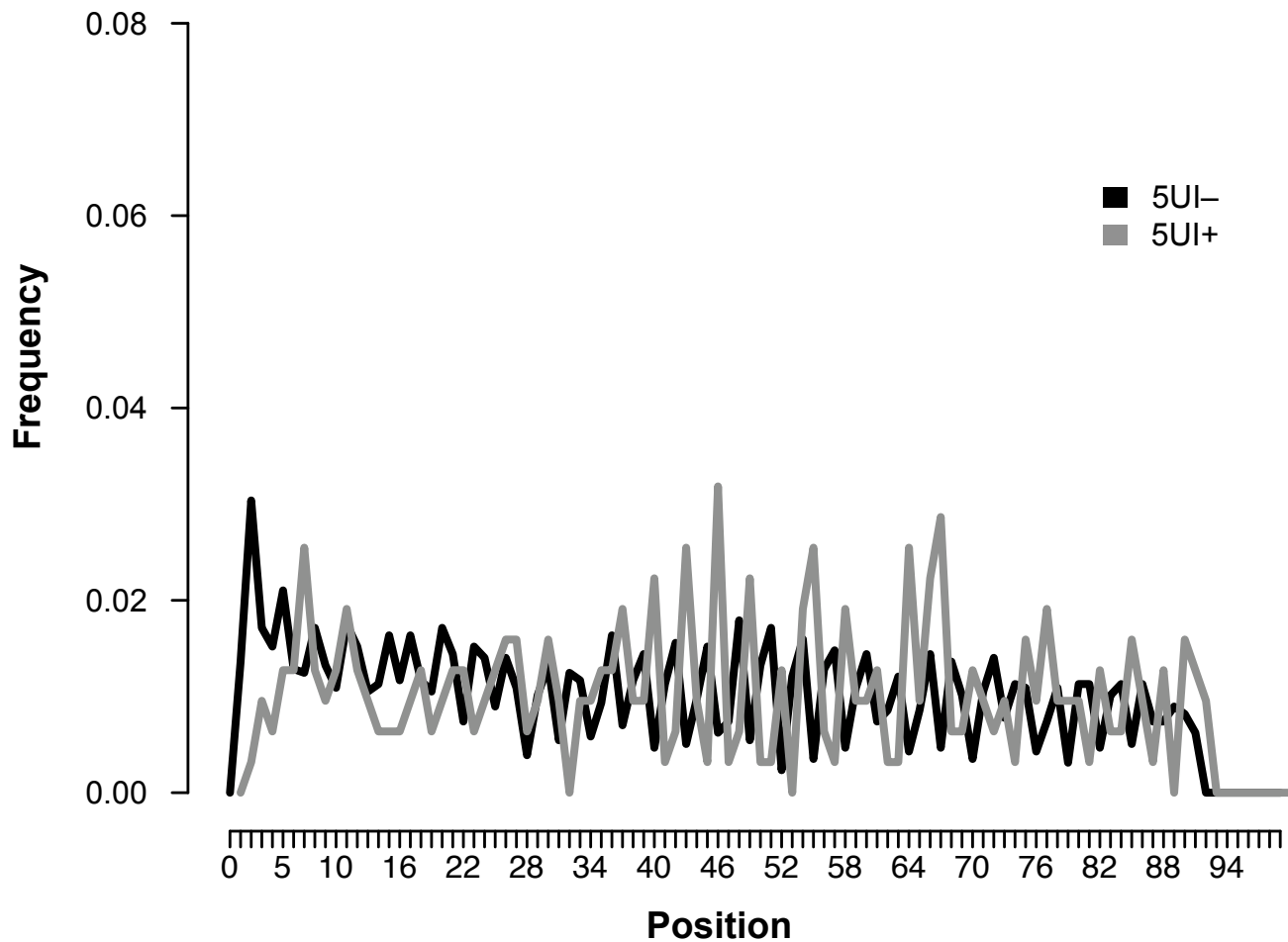

Supplement: Figure S5 — The CGSSGC motif tends to be positioned near the 5′ end among 5UI− genes. The histograms represent the position of all occurrences of the CGSSGC motif. The black line corresponds to fraction of motifs positions among 5UI− genes while the grey line corresponds to that among 5UI+ genes. (0.03 MB PDF) [file pgen.1001366.s006.pdf]

Figure S6

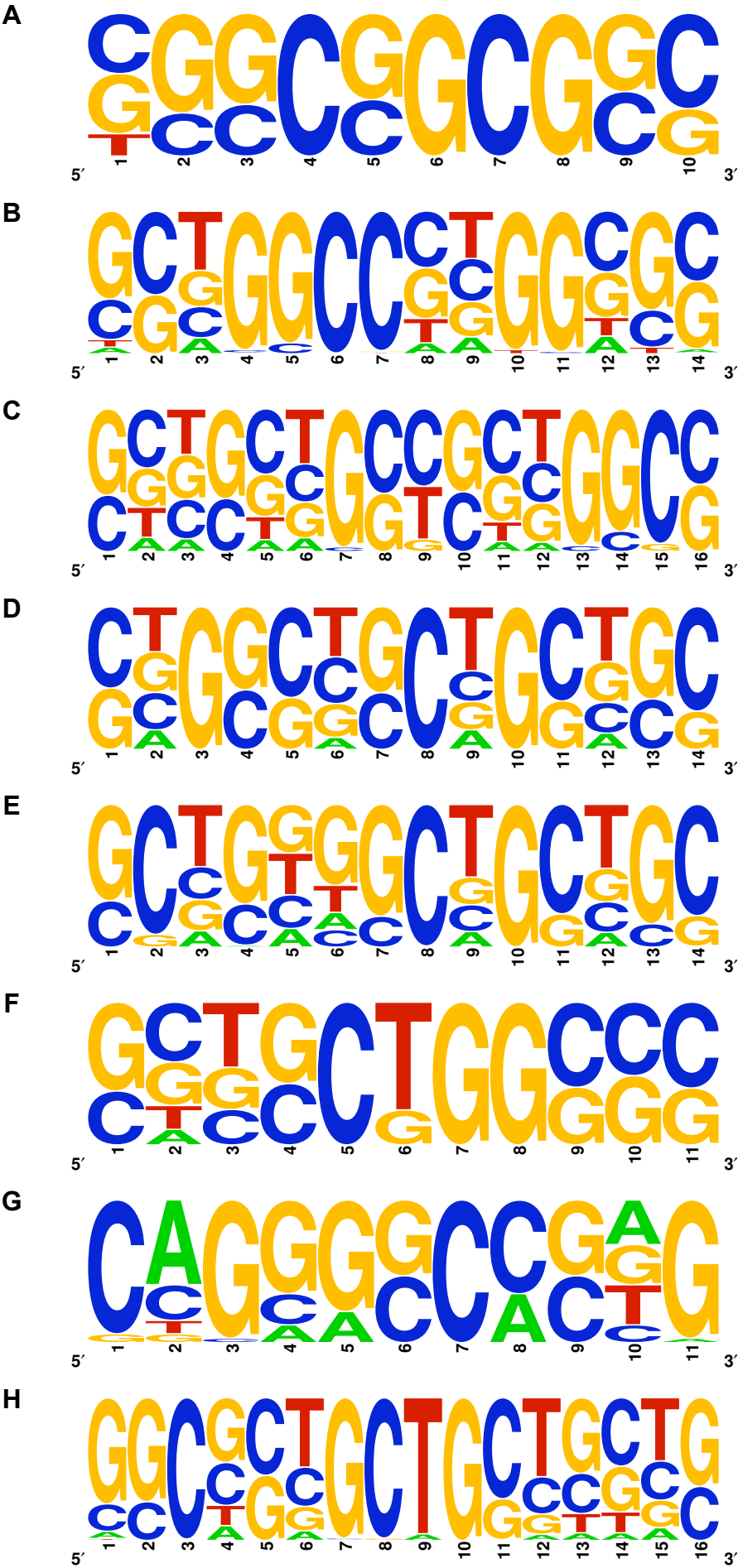

Supplement: Figure S6 — Representation of motifs enriched among SSCR-containing 5UI− genes. WebLogo server [40] was used to visualize the position specific scoring matrices corresponding to eight AlignACE motifs that were most enriched among 5UI− genes. Letter height within each logo reflects the frequency of nucleotides at each position. The panels are in descending order from most enriched motif (panel A) to least enriched motif (panel H) among 5UI− genes. (0.09 MB PDF) [file pgen.1001366.s007.pdf]

Figure S7

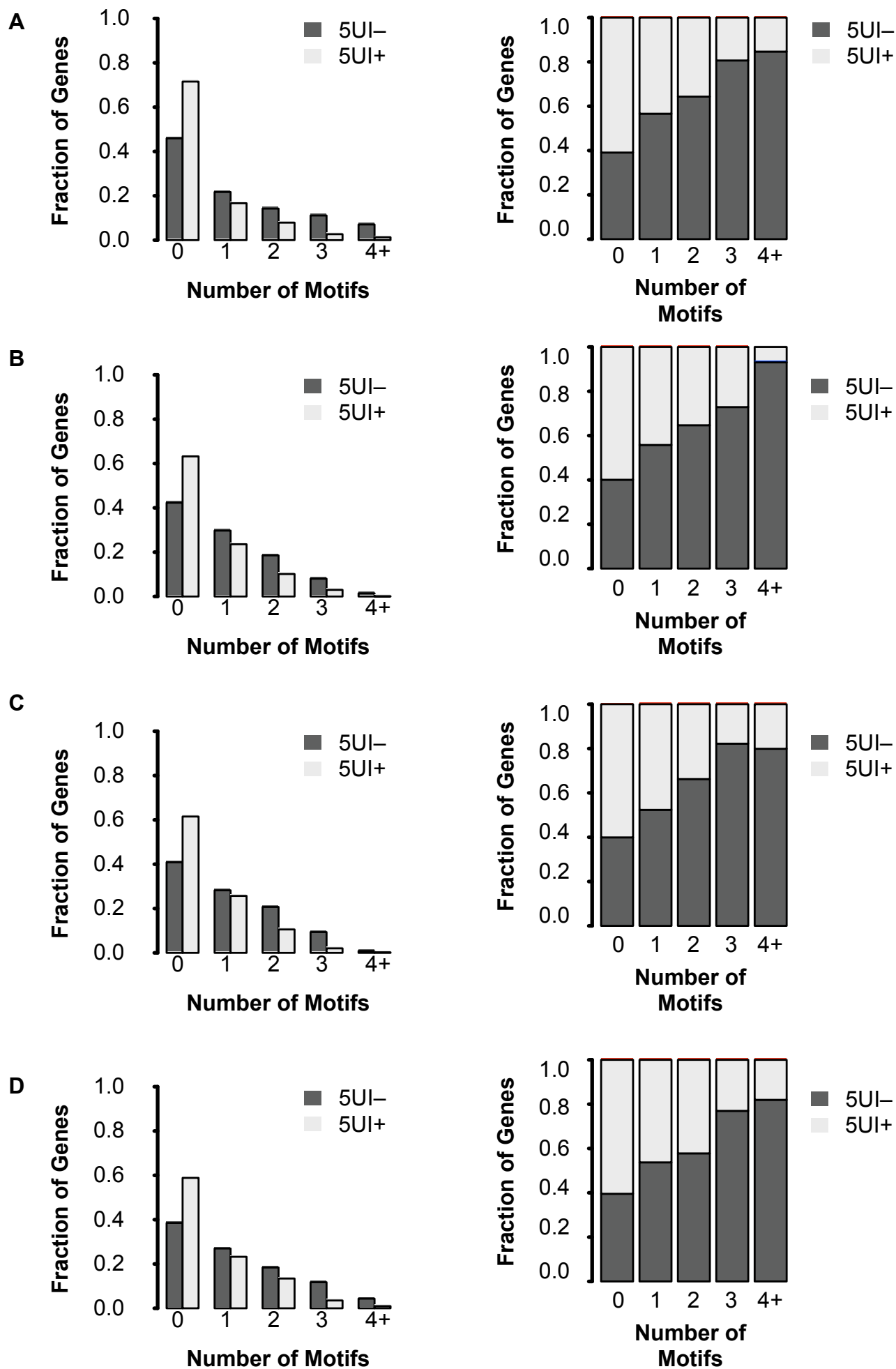

Supplement: Figure S7 — Fraction of genes with one or multiple copies of the discriminative motifs discovered by AlignACE. AlignACE motifs that were most enriched among 5UI− genes are shown in descending order of enrichment (panel A-D). The left panels show the distribution of the number of motifs in the set of SSCR-containing genes with 5UIs (negative set) or without 5UIs (positive set). The right panels show the fraction of sequences in the positive versus negative set for a given number of motif occurrences. For all four motifs shown, the positive set was enriched for the motif, both in terms of fraction of sequences with at least one copy of the motif [(A) 54.1% versus 28.5%; (B) 57.7% versus 36.8%; (C) 59.1% versus 38.5%; and (D) 61.5% versus 41.2%] and in terms of fraction of sequences with multiple motif occurrences [(A) 32.4% versus 11.8%; (B) 28.0% versus 13.2%; (C) 31.0% versus 12.8%; and (D) 34.5% versus 17.9%]. (0.03 MB PDF) [file pgen.1001366.s008.pdf]

**A**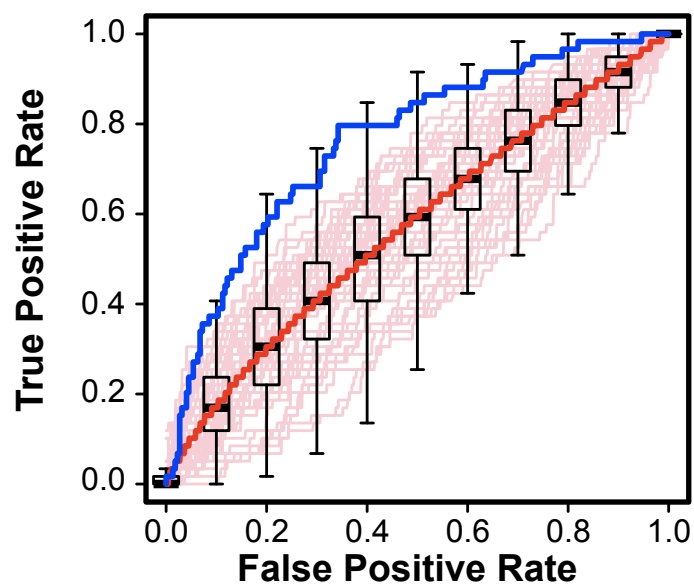**B**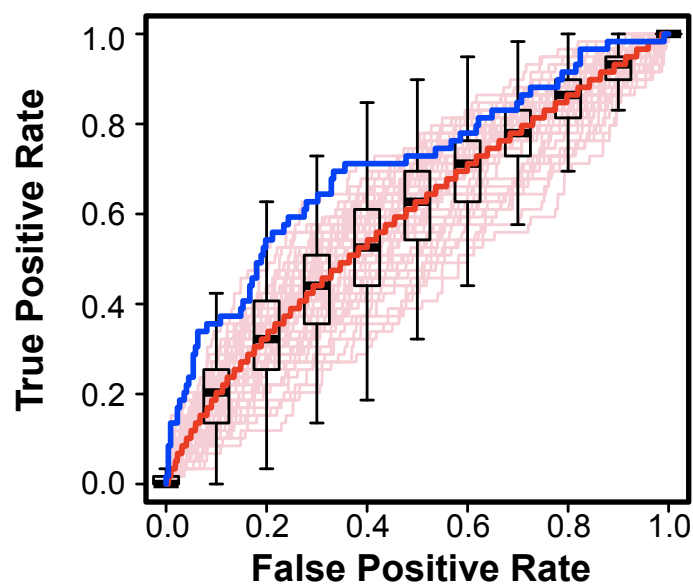**C**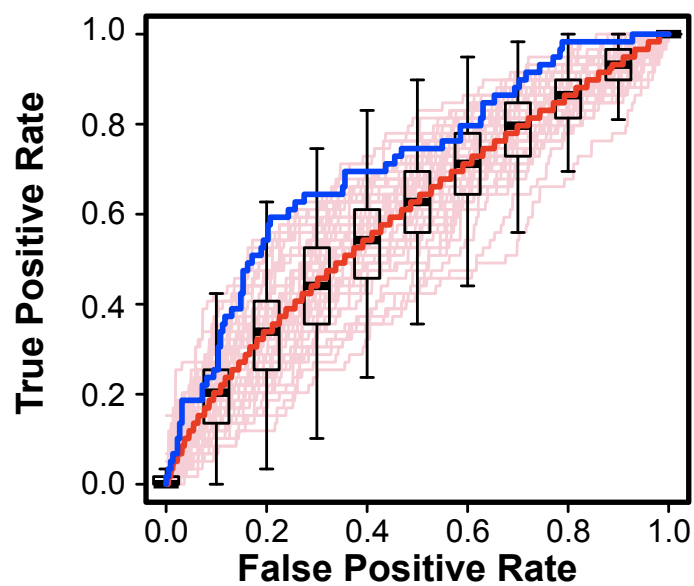**D**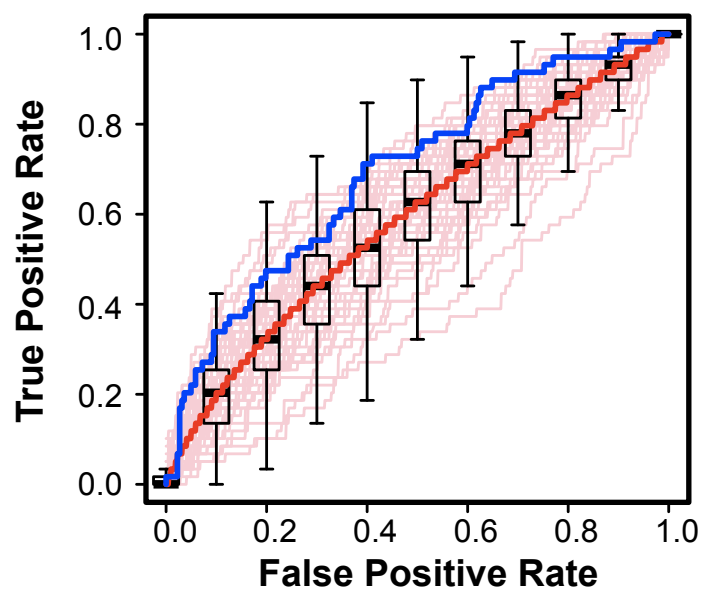

Supplement: Figure S8 — Discriminative motifs discovered by AlignACE are also predictive of 5UI absence among MSCR-containing genes. ROC plots are as described in Figure 6G for the four AlignACE motifs that were most enriched among 5UI− genes in descending order of enrichment (panel A-D). (1.06 MB PDF) [file pgen.1001366.s009.pdf]

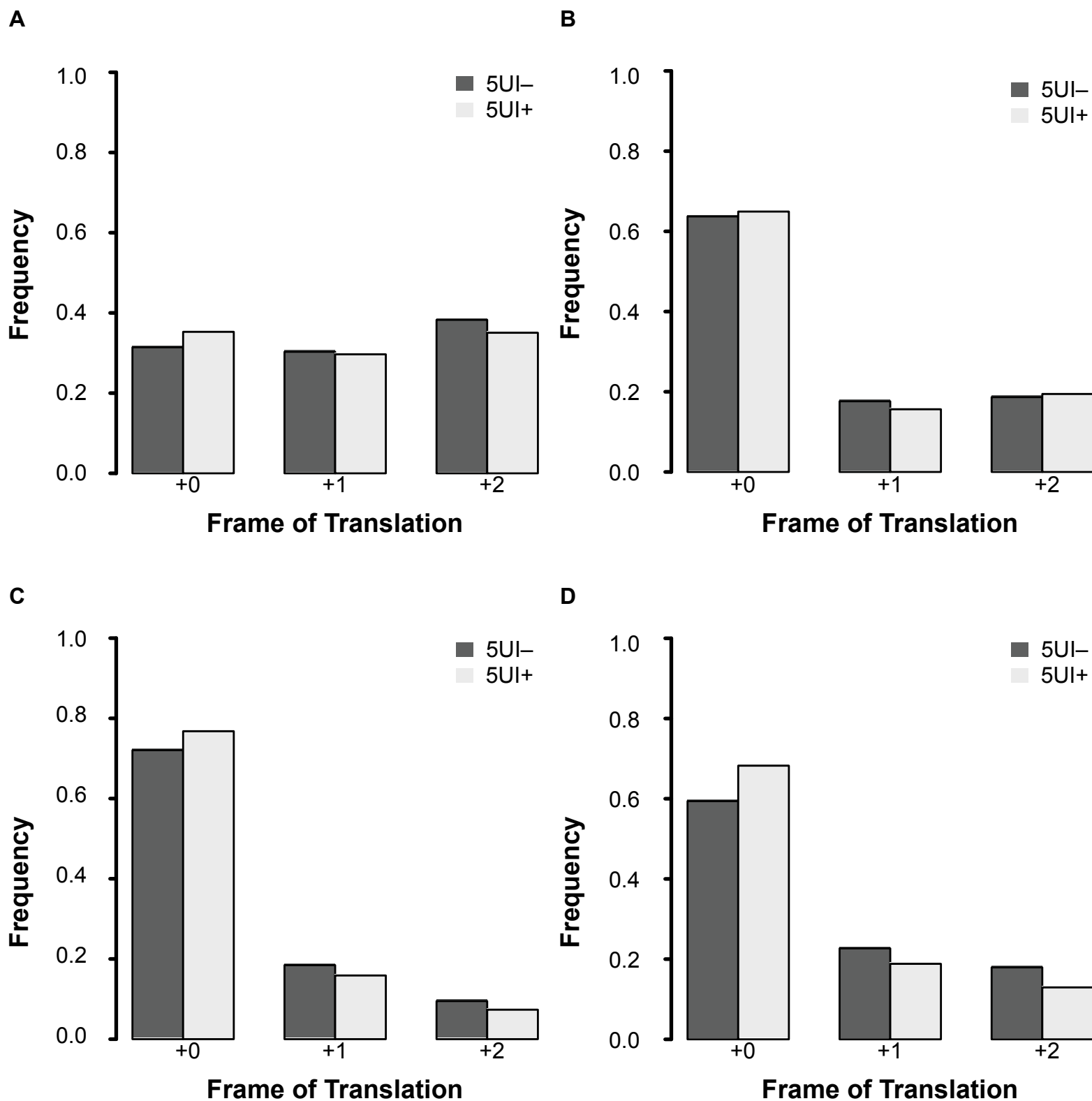

Supplement: Figure S9 — Three of the four discriminative motifs discovered by AlignACE reveal a strong bias for a particular frame of translation. The four AlignACE motifs that were most enriched among 5UI− genes, in descending order of enrichment (panel A-D). The positions of all motif occurrences were classified into one of three possible frames of translation. The fraction of motif occurrences in each frame of translation was plotted for both 5UI− and 5UI+ genes. (0.03 MB PDF) [file pgen.1001366.s010.pdf]
