# Supplementary material for: Genome Analysis Reveals Interplay between 5′UTR Introns and Nuclear mRNA Export for Secretory and Mitochondrial Genes
Source: PLoS Genet. 2011 Apr 14;7(4):e1001366. doi: 10.1371/journal.pgen.1001366 (PMC3077370; doi:10.1371/journal.pgen.1001366)
Supplement: Figure S4 — Nucleotide sequences of experimentally tested SSCRs and MSCRs. Mutations in the PRPΔA and FR7A sequences are indicated in bold. (0.08 MB DOCX) [file pgen.1001366.s005.docx]

*FR-ftz*

ATGGCTTCGCGCTGCTGGCGCTGGTGGGGCTGGTCGGCGTGGCCTCGGACCCGGCTGCCTCCCGCCGGGAGCACCCCGAGCTTCTGCCACCATTT

*FR-7A-ftz*

ATGGC**A**TCGCG**A**TGCTGGCG**A**TGGTGGGG**A**TGGTCGGC**A**TGGCCTCGGACCCG**A**CTGCCTCC**A**GCCGGGAGCACCCCGAGCTTCTGCCACCATTT

*F1-ftz*

ATGCTGTCCGTGCGCGTTGCTGCGGCCGTGGTCCGCGCCCTTCCTCGGCGGGCCGGACTGGTCTCCAGAAATGCTTTGGGTTCATCTTTCATTGC

*PrP-ftz*

ATGGCGAACCTTAGCTACTGGCTGCTGGCACTCTTTGTGGCTATGTGGACTGATGTTGGCCTCTG

*PrP-∆A-ftz*

ATGGCGAACCTT**TC**CT**GG**TGGCTGCTGGC**C**CTCTTTGTGGCT**TGC**TGG**T**C**C**GATGTTGGCCTCTG

*PTH-ftz*

ATGATACCTGCAAAAGACATGGCTAAAGTTATGATTGTCATGTTGGCAATTTGTTTTCTTACAAAATCGGATGG

*MTIF2-ftz*

ATGAACCAGAAGCTACTGAAGTTGGAGAACTTGCTACGATTTCACACTATTTATAGGCAACTGCACAGTCTGTGTCAAAGAAGAGC
