# Supplementary material for: Genome Analysis Reveals Interplay between 5′UTR Introns and Nuclear mRNA Export for Secretory and Mitochondrial Genes
Source: PLoS Genet. 2011 Apr 14;7(4):e1001366. doi: 10.1371/journal.pgen.1001366 (PMC3077370; doi:10.1371/journal.pgen.1001366)
Supplement: Table S1 — 5UIs are depleted from ER-Targeted and Mitochondrial Genes. The GO analysis was done using the FuncAssociate [34], [35]. The functional categories are sorted in descending order based on their log-odds score (LOD). All categories with a LOD score less than -0.1 and p-value< 0.05 are reported. Fisher's Exact Test was used to calculate the statistical significance and correction for multiple hypothesis testing was done using a resampling approach (see [35] for details). (0.07 MB PDF) [file pgen.1001366.s011.pdf]

N	X	LOD	P	P-adj	GO Attribute
5	355	-1.553	3.8e-59	<0.0001	GO:0004984: olfactory receptor activity
0	25	-1.445	1.9e-05	0.0141	GO:0042613: MHC class II protein complex
0	23	-1.409	4.5e-05	0.0339	GO:0002504: antigen processing and presentation of peptide or polysaccharide antigen via MHC class II
0	23	-1.409	4.5e-05	0.0339	GO:0004190: aspartic-type endopeptidase activity
7	313	-1.358	1.8e-48	<0.0001	GO:0007608: sensory perception of smell
2	73	-1.195	1.3e-11	<0.0001	GO:0042611: MHC protein complex
12	337	-1.163	1.6e-46	<0.0001	GO:0007606: sensory perception of chemical stimulus
1	32	-1.059	1.6e-05	0.0114	GO:0045095: keratin filament
2	48	-1.007	3e-07	0.0001	GO:0042612: MHC class I protein complex
5	101	-0.983	3.2e-13	<0.0001	GO:0019882: antigen processing and presentation
2	35	-0.864	4.7e-05	0.0350	GO:0005245: voltage-gated calcium channel activity
14	134	-0.659	3.8e-11	<0.0001	GO:0005882: intermediate filament
14	134	-0.659	3.8e-11	<0.0001	GO:0045111: intermediate filament cytoskeleton
17	133	-0.562	3.7e-09	<0.0001	GO:0004222: metalloendopeptidase activity
9	71	-0.556	1.6e-05	0.0114	GO:0000786: nucleosome
13	89	-0.491	1.1e-05	0.0080	GO:0006334: nucleosome assembly
115	712	-0.464	5.8e-31	<0.0001	GO:0001584: rhodopsin-like receptor activity
17	101	-0.422	3.3e-05	0.0254	GO:0031497: chromatin assembly
123	657	-0.384	1.9e-21	<0.0001	GO:0007600: sensory perception
33	180	-0.383	4e-07	0.0001	GO:0008083: growth factor activity
161	841	-0.375	7.6e-26	<0.0001	GO:0004930: G-protein coupled receptor activity
27	145	-0.373	7.7e-06	0.0050	GO:0009055: electron carrier activity
29	146	-0.339	3.3e-05	0.0257	GO:0020037: heme binding
29	146	-0.339	3.3e-05	0.0257	GO:0046906: tetrapyrrole binding
50	240	-0.316	6.9e-07	0.0002	GO:0005125: cytokine activity
270	1253	-0.315	7.7e-28	<0.0001	GO:0004888: transmembrane receptor activity
202	943	-0.313	1.9e-21	<0.0001	GO:0050877: neurological system process
48	224	-0.300	4.3e-06	0.0027	GO:0008237: metallopeptidase activity
108	497	-0.298	2.2e-11	<0.0001	GO:0005615: extracellular space
242	1056	-0.276	3.1e-19	<0.0001	GO:0007186: G-protein coupled receptor protein signaling pathway
49	216	-0.268	4e-05	0.0302	GO:0008236: serine-type peptidase activity
50	217	-0.260	6.3e-05	0.0441	GO:0017171: serine hydrolase activity
109	466	-0.256	1.4e-08	<0.0001	GO:0004175: endopeptidase activity
72	305	-0.248	6.4e-06	0.0040	GO:0005506: iron ion binding
288	1190	-0.245	1.5e-17	<0.0001	GO:0003008: system process
471	1902	-0.240	2.6e-25	<0.0001	GO:0004872: receptor activity
426	1679	-0.222	6.2e-20	<0.0001	GO:0007166: cell surface receptor linked signal transduction

N	X	LOD	P	P-adj	GO Attribute
194	765	-0.212	1.3e-09	<0.0001	GO:0044421: extracellular region part
210	813	-0.202	2.5e-09	<0.0001	GO:0005739: mitochondrion
196	743	-0.188	8e-08	<0.0001	GO:0005102: receptor binding
640	2360	-0.187	1.3e-19	<0.0001	GO:0004871: signal transducer activity
640	2360	-0.187	1.3e-19	<0.0001	GO:0060089: molecular transducer activity
182	685	-0.183	4.5e-07	0.0001	GO:0008233: peptidase activity
211	782	-0.175	2.7e-07	0.0001	GO:0006508: proteolysis
823	2912	-0.164	1.8e-18	<0.0001	GO:0005886: plasma membrane
172	626	-0.162	1.5e-05	0.0106	GO:0006955: immune response
488	1742	-0.160	8.8e-12	<0.0001	GO:0005576: extracellular region
185	664	-0.154	2.2e-05	0.0155	GO:0015031: protein transport
701	2420	-0.143	1.2e-12	<0.0001	GO:0050896: response to stimulus
229	807	-0.143	1.3e-05	0.0095	GO:0016491: oxidoreductase activity
224	786	-0.141	2.3e-05	0.0168	GO:0033036: macromolecule localization
934	3132	-0.128	1.1e-12	<0.0001	GO:0032501: multicellular organismal process
1495	4899	-0.124	2.6e-16	<0.0001	GO:0016021: integral to membrane
1528	4991	-0.122	4.7e-16	<0.0001	GO:0031224: intrinsic to membrane
1738	5604	-0.116	9.3e-16	<0.0001	GO:0044425: membrane part
2110	6701	-0.112	4.4e-16	<0.0001	GO:0016020: membrane
1210	3898	-0.103	2.7e-10	<0.0001	GO:0007154: cell communication
1108	3565	-0.100	2.9e-09	<0.0001	GO:0007165: signal transduction

LOD score < -0.1 p-value < 0.05
