# Supplementary material for: Genome Analysis Reveals Interplay between 5′UTR Introns and Nuclear mRNA Export for Secretory and Mitochondrial Genes
Source: PLoS Genet. 2011 Apr 14;7(4):e1001366. doi: 10.1371/journal.pgen.1001366 (PMC3077370; doi:10.1371/journal.pgen.1001366)
Supplement: Table S2 — 19 motifs discovered by AlignACE are significantly represented among 5UI− Genes. AlignACE motifs that were enriched among 5UI− genes and the DEME motif were shown. There were 938 and 2594 total sequences in the 5UI+ set and the 5UI− set, respectively. Fisher's exact test was used to test the significance of enrichment of motif containing genes among 5UI− genes. (0.04 MB PDF) [file pgen.1001366.s012.pdf]

ID	CONSENSUS	Fraction of 5UI+ Genes with Motifs	Fraction of 5UI- Genes with Motifs	Fisher's Exact Test p-value (-log10)	Fisher's Exact Test Odds Ratio (log10)
DEME	cggcgc	0.21	0.46	44.28	1.18
1	cggcggcggc	0.28	0.54	42.20	1.09
2	gctggccctggcgc	0.37	0.58	27.91	0.86
3	gctgctgccgctggcc	0.38	0.59	27.07	0.84
4	ctggctgctgctgc	0.42	0.63	26.55	0.83
5	gctggggctgctgc	0.34	0.54	25.25	0.82
6	gctgctggccc	0.13	0.29	23.01	0.99
7	caggggccgag	0.49	0.67	22.80	0.77
8	ggcgctgctgctgctg	0.53	0.71	22.69	0.78
9	cccgggccag	0.51	0.69	21.47	0.75
10	cgcagccccgg	0.56	0.73	21.37	0.77
11	ccggggctgctg	0.45	0.63	20.62	0.73
12	gaggccaggag	0.46	0.63	19.99	0.72
13	gcggaggcgcag	0.38	0.56	19.52	0.71
14	agggcccccagcac	0.40	0.56	16.25	0.64
15	caggaggcgga	0.66	0.79	15.37	0.69
16	gggctgctgg	0.58	0.72	15.11	0.64
17	cagcagcagc	0.75	0.86	13.05	0.71
18	cagccgcaggcacag	0.62	0.75	12.66	0.60
19	cggcgc	0.21	0.46	44.28	1.18
