# Supplementary material for: Genome Analysis Reveals Interplay between 5′UTR Introns and Nuclear mRNA Export for Secretory and Mitochondrial Genes
Source: PLoS Genet. 2011 Apr 14;7(4):e1001366. doi: 10.1371/journal.pgen.1001366 (PMC3077370; doi:10.1371/journal.pgen.1001366)
Supplement: Text S1 — The depletion of 5UIs from SSCRs and MSCRs is not attributable to differences in 5′UTR length. (0.15 MB PDF) [file pgen.1001366.s013.pdf]

Text S1

As genes with short 5'UTRs were less likely to have introns (Supplementary Figure 3A, Wilcoxon rank sum test $p < 1\times10^{-100}$), we tested whether the observed intron depletion in SSCR or MSCR-containing genes could be explained by differences in 5'UTR lengths. We observed that genes in both categories had significantly but only slightly shorter 5'UTRs compared to other genes (Supplementary Figure 3B, Wilcoxon rank sum test $p= 2\times10^{-15}$, $p= 9\times10^{-9}$ for SSCR- and MSCR-containing genes respectively). To test whether these differences explained the depletion, we calculated the posterior expectation of the number of 5'UTR intron-containing genes given the 5'UTR length distribution. We fitted a kernel density estimator to approximate the distribution of the 5'UTR lengths from intron -containing or -lacking genes (Supplementary Figure 3C, 3D). Using these density estimates, for each gene given its 5'UTR length, we calculated the likelihood of having a 5'UTR intron. We, then, used Bayes rule to obtain the posterior likelihood and the posterior probability of 5'UTR intron presence. Using the linearity of expectation, we calculated the expected number of genes with 5'UTR introns in either SSCR- or MSCR-containing genes. In particular, let D_1 and D_0 be the estimated probability density functions of 5'UTR lengths for genes with or without introns respectively. Then,

$$\frac{P(X_i = 1 \mid Y_i = y_i)}{P(X_i = 0 \mid Y_i = y_i)} = \frac{P(Y_i = y_i \mid X_i = 1)}{P(Y_i = y_i \mid X_i = 0)} \frac{P(X_i = 1)}{P(X_i = 0)},$$

where Y_i is the length of the 5'UTR and X_i represents the presence (1) or absence (0) of a 5'UTR intron in the ith gene. We used 0.35/ 0.65 as the ratio of the

prior probabilities ($\frac{P(X_i = 1)}{P(X_i = 0)}$) based on our estimate of the proportion of genes with 5'UTR Introns. The likelihood ratio was calculated by using the estimated densities

$$\frac{P(Y_i = y_i \mid X_i = 1)}{P(Y_i = y_i \mid X_i = 0)} = \frac{D_1(y_i)}{D_0(y_i)}.$$

We define *N* as the random variable that corresponds to the number of genes with 5'UTR introns and by linearity of expectation

$$\mathrm{E}[N] = \sum_i 1 \times P(X_i = 1).$$

Finally, we observed a significant depletion when comparing the observed to the expected number of 5'UTR introns amongst SSCR- or MSCR-containing genes (Fisher's Exact Test $p = 5\times10^{-36}$, and $p = 3\times10^{-6}$ confidence interval of odds ratio 0.59- 0.69 and 0.45-0.73, respectively). This result implied that the observed 5'UTR intron depletion in SSCR- and MSCR- containing genes was not attributable to differences in 5'UTR lengths.
